# SpyCLIP: an easy-to-use and high-throughput compatible CLIP platform for the characterization of protein–RNA interactions with high accuracy

**DOI:** 10.1093/nar/gkz049

**Published:** 2019-01-31

**Authors:** Ya Zhao, Yao Zhang, Yilan Teng, Kai Liu, Yanqing Liu, Weihua Li, Ligang Wu

**Affiliations:** 1 State Key Laboratory of Molecular Biology, Shanghai Key Laboratory of Molecular Andrology, CAS Center for Excellence in Molecular Cell Science, Institute of Biochemistry and Cell Biology, Chinese Academy of Sciences; University of Chinese Academy of Sciences, Shanghai, China; 2 Institute of Translational Medicine, School of Medicine, Yangzhou University, Yangzhou, China; 3 Jiangsu Key Laboratory of Experimental & Translational Non-coding RNA Research, Yangzhou University, Yangzhou, China; 4 NHC key Laboratory of Reproduction Regulation (Shanghai Institute of Planned Parenthood Research), pharmacy school, Fudan University, Shanghai, China; 5 School of Life Sciences, Shanghai University, Shanghai, China; 6 Institute of Integrated Traditional Chinese and Western Medicine, School of Medicine, Yangzhou University, Yangzhou, China; 7 NHC key Laboratory of Reproduction Regulation (Shanghai Institute of Planned Parenthood Research), Fudan University, Shanghai, China

## Abstract

UV crosslinking and immunoprecipitation (CLIP) coupled with high-throughput sequencing is the most state-of-the-art technology to characterize protein–RNA interactions, yet only a small portion of RNA-binding proteins (RBPs) have been studied by CLIP due to its complex procedures and high level of false-positive signals. Herein, we report a SpyCLIP method that employs a covalent linkage formed between the RBP-fused SpyTag and SpyCatcher, which can withstand the harshest washing conditions for removing nonspecific interactions. Moreover, SpyCLIP circumvents the radioactive labeling and PAGE-membrane purification steps, and the whole procedure can be performed on beads and is readily amenable to automation. We investigated multiple RBPs by SpyCLIP and generated high-quality RNA binding maps with significantly improved reproductivity and accuracy. Therefore, the small tag size and convenient protocol of SpyCLIP provides a robust method for both routine characterization and high-throughput studies of protein–RNA interactions.

## INTRODUCTION

Throughout their lifetime, cellular RNAs rarely exist as naked molecules but form ribonucleoprotein (RNP) complexes with omnipresent partners called RNA-binding proteins (RBPs) (1–3). Mounting evidence indicates that RNPs play a major role in both normal and pathological processes in cells (4–6). UV crosslinking and immunoprecipitation (CLIP) coupled with high-throughput sequencing (-seq) is one of the most powerful technologies to precisely identify *in vivo* protein–RNA interactions (7,8). Several major varieties, such as photoactivatable-ribonucleoside-enhanced CLIP (PAR-CLIP) (9), individual-nucleotide-resolution CLIP (iCLIP) (10), enhanced CLIP (eCLIP) (11) and infrared-CLIP (irCLIP) (12), have been developed to improve the performance and reduce the technical difficulty of this technology. However, the current CLIP methods rely on the specific interaction between antibodies and antigens. Due to limited antibody-antigen affinity, RNPs cannot withstand harsh washing conditions, and thus, the purity of RNPs after immunoprecipitation (IP) remains insufficient. Consequently, SDS-PAGE, followed by the transfer of RNPs onto the nitrocellulose membrane and RNA recovery from the membrane are routinely performed to further purify RNPs. Although such procedures increase the specificity of the obtained data, the loss of copurified RNAs during membrane transfer and RNA recovery is tremendous. Moreover, the requirement of gel electrophoresis and membrane transfer to purify RNPs has strongly hindered high-throughput applications of CLIP technology.

We propose that a stronger interaction, ideally a covalent linkage, formed between the RBPs and the purification matrix would allow much more stringent washing conditions during RNP purification, thereby circumventing the need for gel purification steps without sacrificing the purity of RNPs. The CRAC technique first introduced denaturing washing conditions with 6 M guanidine hydrochloride during RNP purification, which employed a tandem affinity purification tag that consisted of a 6 × His tag, a TEV cutting site and a 2 × Protein A (ProtA) tag (13). However, the CRAC method did not employ covalent linkage between the RBP and the beads, which could only tolerate limited denaturing conditions and would inevitably have higher nonspecific binding, including endogenous proteins that have histidine clusters, to the nickel beads and still required gel purification steps. The recently developed uvCLAP method (14) used another tandem affinity purification tag that consisted of two 6 × His tags and an *in vivo* biotinylation signal peptide, which allowed tagged RBPs to be sequentially purified by nickel and streptavidin beads. The uvCLAP method applied stringent washing conditions during the streptavidin pull-down step and omitted the gel purification of the RNP. Nevertheless, due to the noncovalent nature of the biotin-streptavidin interaction, denaturing washing conditions cannot be applied to uvCLAP and hence limits the purity of the RNP with potential contamination of residual endogenous histidine-rich proteins and endogenous biotinylated proteins. More recently, GoldCLIP (15) utilized the commercially available HaloTag-HaloLink covalent purification system to achieve the denaturing purification of HaloTag-fused RBPs, which successfully omitted the gel purification steps. However, HaloTag is a 33-kilodalton (kDa) protein tag, which may influence RBP localization and function and is also difficult to incorporate into endogenous genes.

Herein, we developed a SpyTag-based CLIP (SpyCLIP) method based on the covalent SpyTag-SpyCatcher system to allow denaturing washing conditions, thereby completely circumventing the rate-limiting steps of the traditional CLIP technique, including the generation of highly specific antibodies, gel electrophoresis and membrane transfer of RNP, as well as the recovery of RNA from the membrane. Importantly, the SpyTag fused to the RBP is a very small peptide that consists of only 13 amino acids (aa), which not only ensures a minimal influence on RBP activity but is also readily amenable to gene editing approaches, thereby permitting studies of endogenous RBPs by SpyCLIP in animal models. We also redesign the library construction strategy that allows all of the reaction steps to be continuously performed on beads or in solution. These improvements not only dramatically reduce the technical difficulty and procedure time of the CLIP technique without scarifying quality but also make it readily amenable to automation and high-throughput applications. In addition, we introduce a single universal input control to remove background noise, further guaranteeing excellent specificity in identifying authentic binding sites. Using this new tool, we generated highly complex and reproducible RNA binding maps for diverse well-studied RBPs with outstanding sensitivity and specificity and revealed novel insights into their functional mechanisms, demonstrating that SpyCLIP represents an easy-to-use and highly accurate tool for systematic studies of protein–RNA interactions.

## MATERIALS AND METHODS

### Plasmid construction

The full-length open reading frame (ORF) of SpyCatcher (GenBank ID: JQ478411) was chemically synthesized (Synbio Technologies, Suzhou, Jiangsu, China). An 8-lysine repeat and a 3 × (GGGGS) linker were attached to the C-terminus of SpyCatcher ORF by overlap PCR. The modified SpyCatcher ORF was then cloned into the pET-28a (Novagen, Madison, Wisconsin, United States) expression vector containing a 6 × His tag at the N-terminus of the expression cassette through Nco I and Xho I restriction sites. The Doxycycline (Dox)-inducible lentiviral vector pTRIPZ (Dharmacon, Lafayette, Colorado, United States) was modified to incorporate N-terminal sequences encoding a 3 × FLAG tag (DYKDHDGDYKDHDIDYKDDDDK), a PreScission Protease site (LEVLFQGP), and a SpyTag (AHIVMVDAYKPTK). The ORF of SLBP, RBFOX2 and AGO2 were PCR-amplified from the cDNAs of HEK293T cells and inserted downstream of the SpyTag sequence of the modified pTRIPZ vector via Age I and Pac I restriction sites.

### Cell culture and stable cell lines

Lenti-X 293T (Clontech, Mountain View, California, United States) cells were maintained in Dulbecco's modified Eagle's medium (Clontech, Mountain View, CA, USA) with 10% fetal bovine serum (Gibco, Chagrin Falls, OH, USA). To produce the lentiviruses, Lenti-X 293T cells were transfected with a virus vector encoding the expression cassette as well as the VSVG and ΔR8.91 plasmids. Viruses were harvested 48 h post-transfection. RBP-expressing Lenti-X 293T stable cell lines were generated by transduction with lentiviruses in the presence of 8 μg/ml of polybrene (Sigma-aldrich, St. Louis, MO, USA) overnight, followed by selection with 1 μg/ml puromycin (BBI, Shanghai, China) for 1 week. To induce RBP expression, 1 ng/ml Dox (BBI, Shanghai, China) was added to the cell culture medium, and the cells were harvested 48 h after induction. To induce acute translation suppression, AGO2-expressing Lenti-X 293T stable cells were treated with 2 μg/ml harringtonine (HAR) (MCE, Monmouth Junction, NJ, USA) for 1 h at 37°C before harvesting.

### Protein expression and purification

*Escherichia coli* Rosetta(DE3)pLysS competent cells (Novagen, Madison, Wisconsin, United States) were transformed with SpyCatcher expression plasmids. A single colony was picked and inoculated into 2 ml Luria-Bertani (LB) medium containing 0.5 mg/ml kanamycin (BBI, Shanghai, China) overnight at 37°C. Overnight cultures were diluted 100-fold and grown at 37°C until the OD600 reached 0.5, followed by induction with 1 mM isopropyl β-d-thiogalactoside (IPTG) (BBI, Shanghai, China) at 30°C for 4 h. Cells were harvested and lysed using a high-pressure homogenizer (Uion-Biotech, Shanghai, China) in His binding buffer (20 mM phosphate buffer pH 7.8, 500 mM NaCl, 20 mM imidazole,1 mM PMSF). The lysates were centrifuged at 15 000 rpm for 50 min, and the soluble fraction was loaded onto a column packed with Ni Sepharose (GE Healthcare, Chicago, IL, USA). The column was washed sequentially with 10 column volumes (CVs) of His binding buffer, 5 CVs of low-salt wash buffer (20 mM phosphate buffer pH 7.8, 150 mM NaCl), 10 CVs of high-salt wash buffer (20 mM phosphate buffer pH 7.8, 2 M NaCl), and another 5 CVs of low-salt wash buffer, and finally eluted with 7 CVs of elution buffer (20 mM phosphate buffer pH 7.8, 500 mM NaCl, 150 mM imidazole). The eluates were diluted to a final concentration of 100 mM NaCl and loaded onto an anion ion-exchange column (GE Healthcare, Chicago, IL, USA), and the fractions were collected using the ÄKTA start instrument. The purity of each fraction was checked by SDS-PAGE and Coomassie blue staining. Fractions that exhibited >95% purity were combined and concentrated using a 10-kDa molecular mass cut-off centrifugal filter unit. Purified SpyCatcher proteins (∼8 mg/ml in PBS) were aliquoted and stored at –80°C.

### SpyCatcher magnetic bead preparation

For a single SpyCLIP experiment, 1 mg of Epoxy beads (1 μm diameter, BioMag, Wuxi, Jiangsu, China) was washed three times with 1 ml of PBS and re-suspended in 50 μl PBS containing 0.1 mg SpyCatcher proteins. After overnight rotation at 25°C, the beads were washed three times with 1 ml of Spybeads wash buffer (PBS containing 500 mM NaCl and 0.5% Triton X-100) and then blocked with 1 ml of Spybeads blocking buffer (PBS containing 1% BSA and 0.1% Triton X-100) for 6 h at 25°C. The SpyCatcher-coupled beads were stored at 4°C until use.

### Real-Time quantitative PCR

RBP-expressing Lenti-X 293T stable cells (1 × 10^7^) were lysed with 1 ml of SpyCLIP lysis buffer (50 mM Tris pH 7.4, 150 mM NaCl, 0.5% sodium deoxycholate, 0.1% SDS, 1% Triton X-100). Total RNA was extracted from 100 μl of the lysate, dissolved in 10 μl H_2_O and saved as the input sample. The remaining lysate was equally divided and subjected to FLAG and IgG IP. RNA was extracted after IP, dissolved in 10 μl H_2_O and saved as the IP sample. Input and IP RNA samples were treated with 2 U Turbo DNase (Invitrogen, Carlsbad, CA, USA) and then reverse transcribed using M-MLV (Beyotime, Nantong, Jiangsu, China), according to the manufacturer's instructions. The RT products were diluted 10-fold and subjected to quantitative real-time PCR. Real-time PCR was performed on a StepOnePlus real-time PCR system (Applied Biosystems, Foster City, CA, USA) with SYBR Green PCR master mix. The PCR mixtures were heated to 95°C for 1 min and then subjected to 40 amplification cycles (10 s at 95°C, 15 s at 55°C, 30 s at 72°C). qPCR primers were as follows:

*ENAH*: 5′-CGCTCCACTTCCTGAGTAT-3′, 5′-GCTGAGATGACTTTAGCGTAT-3′.
*CENPI*: 5′-GCAGGCTACAGTCAAACC-3′, 5′-GTCAAGGAGCGGCAAGAA-3′.
*HM13*: 5′-AATGCTATTGTGCCCTCA-3′, 5′-GCACCAAGCAGGTAAAGG-3′.
*PKM*: 5′-GAGGCAGCGTGGTCAAGT-3′, 5′-GAGACAATCTGGCAGTAGG-3′.
*SMG7*: 5′-AGTCTGGCTGCCTTGGAG-3′, 5′-CCAGCAGAGCCGGGTAAA-3′.
*SRRM2*: 5′-GGGGTCCTTGGTTTCTTC-3′, 5′-ACCAGAGCCCTGGCAGTA-3′.
*RPL34*: 5′-CCGAACCCCTGGTAATAGA-3′, 5′-ACCCCTCGAAGTCTGCCTG-3′
*PRDX3*: 5′-CATCCCTGAAATTGAGTTT-3′, 5′-CTGGATTTGATAAATATCCTT-3′.

### Immunofluorescence

Dox-induced Lenti-X 293T cells were fixed in 4% paraformaldehyde diluted in PBS for 15 min and permeabilized with 0.3% Triton X-100 in PBS for 20 min at room temperature. The cells were blocked with 1% BSA and 0.1% Triton X-100 in PBS for 30 min at room temperature. The cells were incubated with anti-FLAG (1:1000) (Sigma-Aldrich, St. Louis, MO, USA) and anti-PTB (1:250) (Proteintech, Rosemont, IL, USA) antibodies for 1 h at room temperature in PBS containing 0.1% Triton X-100, respectively. After washing with PBS, the cells were incubated with CY3- or Alexa 488-labeled secondary antibodies (1:1000) (Molecular probes, Eugene, OR, USA) with the appropriate serotype for 1 h at room temperature. Hoechst 33342 (1:1000) (Sigma-Aldrich, St. Louis, MO, USA) was applied to stain DNA at room temperature for 10 min. The cells were blocked in fluorescent mounting medium (DaKo, Carpinteria, CA, USA) and imaged with a microscope.

### SpyCLIP RNP purification and library construction

See Supplementary Figures and Protocols.

### High-throughput sequencing and mapping

High-throughput sequencing of SpyCLIP libraries was performed on the Illumina HiSeq X10 platform. The unique molecular identifier (UMI, 10-nt random sequence) added to the 5′ end of each read during library construction was removed and used as the read identity (ID) in the following analysis. The adapter sequence at the 3′ end of the sequenced reads was removed with the FASTX program (version 0.0.14) (-l 1 -c). Insert cDNAs that were either too short (<30 nt) or too long (>65 nt) were discarded. The remaining reads were mapped to the human genome (version hg38) using the STAR program with –outFilterMultimapScoreRange 1 –outFilterScoreMin 10 –outFilterMultimapNmax 1 (version 2.5.2b). Reads that mapped to the same genomic position and had identical UMIs were categorized as PCR duplicates and then collapsed into a single read. All remaining reads after processing were regarded as usable reads and used to identify clusters. Mapped reads were visualized on the Integrative Genomics Viewer (IGV). To check the saturation point of data generated from SpyCLIP, different numbers (from 1 to 20 million) of uniquely mapped reads were randomly selected to calculate the usable read ratio at different analogic sequencing depths.

### Identification of SpyCLIP clusters and noise deduction

Because the 3′ end of the insert cDNA was the RBP crosslinking site, the clusters were identified based on the 5′ start position of usable reads using the iCount program with a 3-nt clustering window. The number of usable reads in each cluster was normalized to RPM (reads per million genome mapped reads) and considered as the cluster abundance. The reproducibility of SpyCLIP was determined by comparing the abundances of all identified clusters in biological and technical replicate samples. To remove noise signals, SpyCLIP clusters were normalized against input clusters. The abundance of each cluster in SpyCLIP and input sample was calculated independently. SpyCLIP clusters exhibiting at least 15-fold enrichment over their corresponding input counterparts were identified as RBP-specific clusters.

### Annotation of RBP-specific clusters

The genomic annotation of human histone genes and gene elements was downloaded from GENCODE (version 27). The PTBP1-specific clusters were classified into four categories with the following order: intron, 3′ untranslated region (UTR), coding sequence (CDS), and 5′ UTR. To identify the motifs bound by PTBP1, pentamers enriched in the region [–10 to +10] around the summit of each cluster were detected. The pentamers with an enrichment *z*-score ≥200, compared to randomly chosen regions of the same size from the same annotation class (intron, 3′ UTR, CDS or 5′ UTR), were used as PTBP1-binding pentamers for the downstream analysis. The RBFOX2-specific clusters were classified into the same four categories as PTBP1-specific clusters. The 80-nt region centered around the summit of each cluster was extracted and used to identify the *de novo* RBFOX2 binding motif using HOMER’s findMotifs program (-p 4 -rna -S 10 -len 6). For SLBP-specific clusters, the genomic overlapping status of each cluster and known histone genes was annotated. For AGO2-specific clusters, each cluster was annotated to be associated with a protein-coding gene or long noncoding RNA (lncRNA) based on its genomic overlapping status. The clusters overlapping with protein-coding genes were then classified into four categories with the following order: CDS, 3′ UTR, 5′ UTR, and intron. The number of clusters classified into each category was counted for comparison among the samples.

### Visualization of RBP-specific cluster distributions around the RBP-regulated exons

The RNA sequencing data upon knockdown of PTBP1/PTBP2 and RBFOX2 in HEK293 cells were downloaded through the GEO accession numbers GSE69656 and GSE60392. The sequencing reads were mapped to the human genome (version hg38) using the STAR program with –outFilterMultimapScoreRange 1 –outFilterScoreMin 10 –outFilterMultimapNmax 1 (version 2.5.2b). The identification of alternative splicing events was performed by using the rMATS program with default parameters. The enrichment of RBP-specific clusters near the regulated exons was calculated using the code download at https://github.com/jernejule/clip-data-science.

### Identification of HAR-enriched AGO2 clusters

The identified AGO2-specific clusters in wild type (WT) and HAR conditions were combined, and the abundance of each cluster in WT or HAR condition was calculated respectively. Clusters in HAR condition that exhibited at least a 2-fold increase over their counterparts in WT condition were defined as HAR enriched.

### Distribution of AGO2 SpyCLIP reads along transcripts

The reads in AGO2-specific clusters were remapped to protein-coding or lncRNA transcripts. To normalize the length of different transcripts, 5′ UTRs, CDSs and 3′ UTRs of protein-coding genes were divided into 50 parts, and lncRNA transcripts were divided into 150 parts. The number of usable reads mapped to different parts of each gene was counted, and the summarized results for different genes was used to estimate the distribution.

### MicroRNA (miRNA) expression detected by AGO2 IP

The adapter sequences were removed from the 3′ end of the sequenced reads using the FASTX program (version 0.0.14)(-l 1 -c). The miRNA expression level was determined by the number of reads that mapped to the mature miRNA sequences annotated in miRbase (version 21), allowing –2 or +2 nt to be templated by the corresponding genomic sequence at the 3′ end.

### Prediction of miRNA target sites within AGO2 SpyCLIP clusters

The top 50 or top 100 miRNAs identified by AGO2 IP were selected, and successive 7-mer sequences along each miRNA were used to search for complementary targets in AGO2 SpyCLIP clusters. These 7-mer sequences were classified by the starting position of the miRNA. The minimum free energy for the hybridization of a full-length miRNA and its corresponding target sequence was calculated using the RNAhybrid program.

## RESULTS

### Development of a covalent linkage-based SpyCLIP method

**Figure 1.**
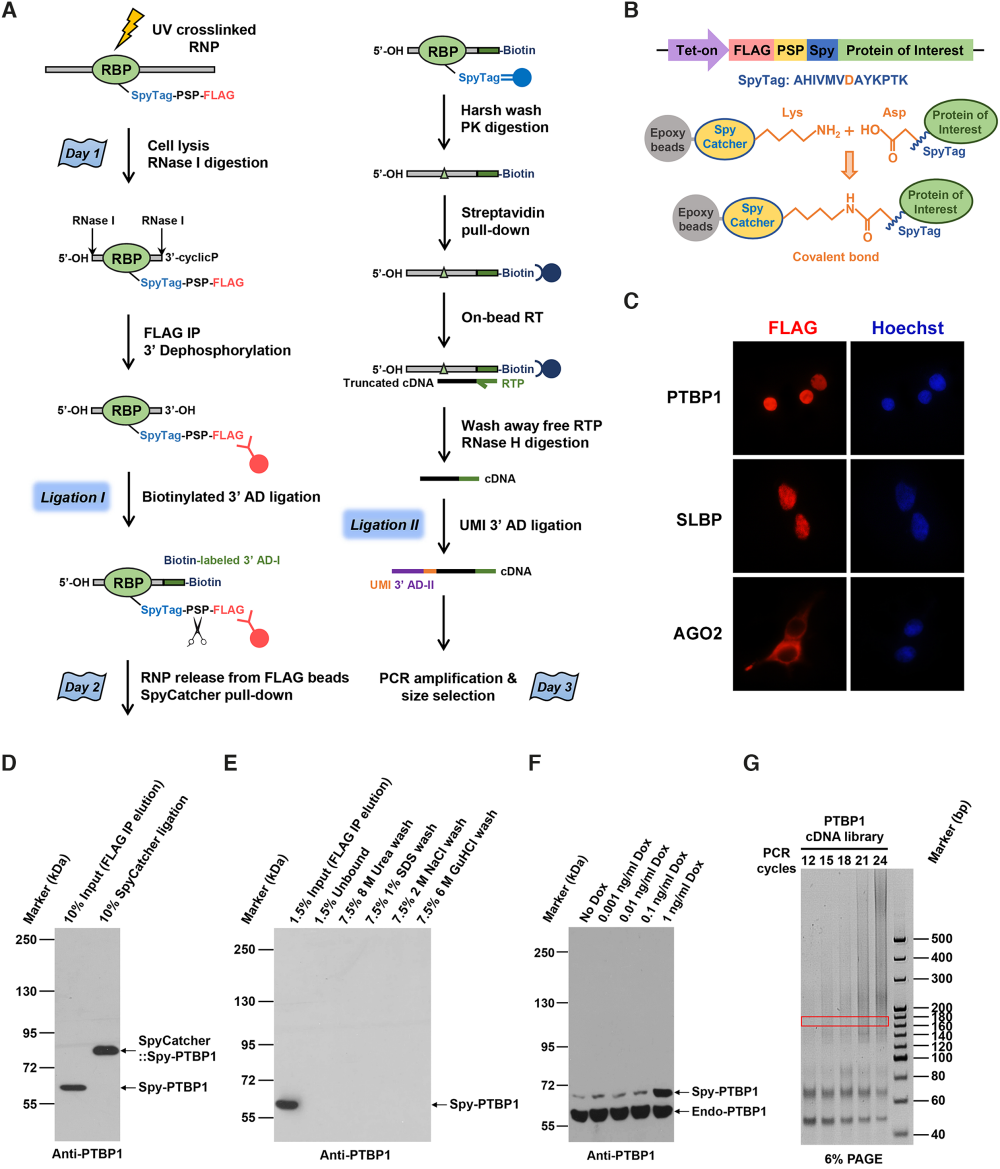
A highly efficient covalent linkage-based SpyCLIP platform for the characterization of protein–RNA interactions. (**A**) Schematic representation of the SpyCLIP procedure. (**B**) Schematic representation of the Spy-tagged RBP structure and mechanism of the SpyTag-SpyCatcher covalent interaction. (**C**) Cellular localization of Spy-tagged RBPs ectopically expressed in Lenti-X 293T cells. (**D**) The modified SpyCatcher protein efficiently formed a stable complex with Spy-tagged PTBP1 in SpyCLIP lysis buffer. (**E**) The covalent SpyTag-SpyCatcher pull-down system can withstand harsh washing conditions, including buffers containing 8 M urea, 1% SDS, 2 M NaCl or 6 M guanidine hydrochloride. (**F**) Comparison of expression levels of Dox-induced Spy-tagged PTBP1 with its endogenous counterpart in Lenti-X 293T cells, and 1 ng/ml Dox was used for all RBPs used in this study. The PTBP1 band in the No Dox lane is due to leak expression of the tet-on system. (**G**) Amplification of PTBP1 SpyCLIP libraries using different PCR cycles. Twenty PCR cycles can provide a sufficient amount of cDNA for deep sequencing in a relatively small volume and did not increase the percentage of PCR duplicates; hence, 20 PCR cycles were chosen for library preparations. The region marked by the rectangle (160–180 bp) was recovered for deep sequencing.

The FbaB protein of bacterial *Streptococcus pyogenes* contains a CnaB2 domain that can form a spontaneous intramolecular isopeptide bond (16). This domain can be split into a 13-aa SpyTag peptide and a 12-kDa SpyCatcher protein. The Asp residue of the SpyTag peptide would rapidly form a covalent bond with the Lys residue of the SpyCatcher protein when the SpyTag peptide and SpyCatcher protein come into contact via a highly specific reaction (16) (Figure. 1B). Taking advantage of the highly reactive SpyTag-SpyCatcher chemistry, we developed a gel-free CLIP method with a significantly improved signal-to-noise ratio, named SpyCLIP (Figure 1A). We fused the RBP of interest to a SpyTag sequence together with a FLAG-Tag. The tag is small and does not interfere with the cellular localization of the RBPs we tested, such as PTBP1, SLBP and AGO2 (Figure 1C). After enrichment by anti-FLAG beads, the RNP was released into the solution and then remixed with magnetic beads coated with the SpyCatcher protein (Supplementary Figure S1A). The SpyTag-fused RBP rapidly attached to the SpyCatcher beads via an irreversible covalent linkage (Figure 1D and Supplementary Figure S1B) and could withstand extremely stringent washing conditions, including the application of buffers containing 8 M urea, 1% SDS, 2 M NaCl and 6 M guanidine hydrochloride (Figure 1E and Supplementary Figure S1C). Such a stringent purification scheme greatly increases the purity of the RNP and thus completely omits any PAGE-membrane purification steps required by most CLIP methods, making SpyCLIP readily amenable to automation and high-throughput applications.

To further simplify the SpyCLIP procedure without sacrificing sensitivity and specificity, we redesigned the library construction strategy (Figure 1A). Briefly, UV crosslinked cells were lysed under mild conditions, and RBP-bound RNAs were partially digested with RNase I, which showed no obvious nucleotide bias. The tagged RBP was enriched from the cell lysate by FLAG IP, and a 3′-biotinylated DNA adapter was ligated to the 3′ end of the captured RNA fragments. The ligated RNPs were released into the solution by PreScission Protease cleavage and then covalently coupled to the SpyCatcher beads. The covalently linked RNPs were extensively washed under denaturing conditions to remove any residual proteins and RNA bound nonspecifically. The RNA fragments linked to biotinylated 3′ adaptors were released from RBPs by proteinase K digestion and captured by streptavidin beads. After reverse transcription (RT), the cDNAs were ligated to a second DNA adapter containing a unique molecular identifier (UMI-adapter), and the resultant cDNA library was ready for PCR amplification and deep sequencing (Figure 1G and Supplementary Figure S2C). Because RT reactions often stop at the crosslinking site (17), the sequence next to the UMI-adapter ligation site represented *in vivo* RBP binding sites, thus avoiding sophisticated statistical analyses.

The entire SpyCLIP procedure requires only one and a half days of hands-on time and demonstrates a high success rate, representing the most user-friendly CLIP method so far. This procedure completely avoids the SDS-PAGE separation, membrane transfer of RNPs and RNA labeling for the visualization and recovery of RNA from the membrane, thus minimizing the risk of RNA loss during PAGE-membrane purification steps and ensuring high complexity and reproducibility of the obtained libraries. More importantly, these improvements overcome the intrinsic defects of current gel-dependent CLIP methods and make the CLIP technology suitable for automation and high-throughput applications, which would enable large-scale profiling of numerous RBPs in a cost-effective way.

### SpyCLIP generates high-quality and reproducible RNA binding maps

**Figure 2.**
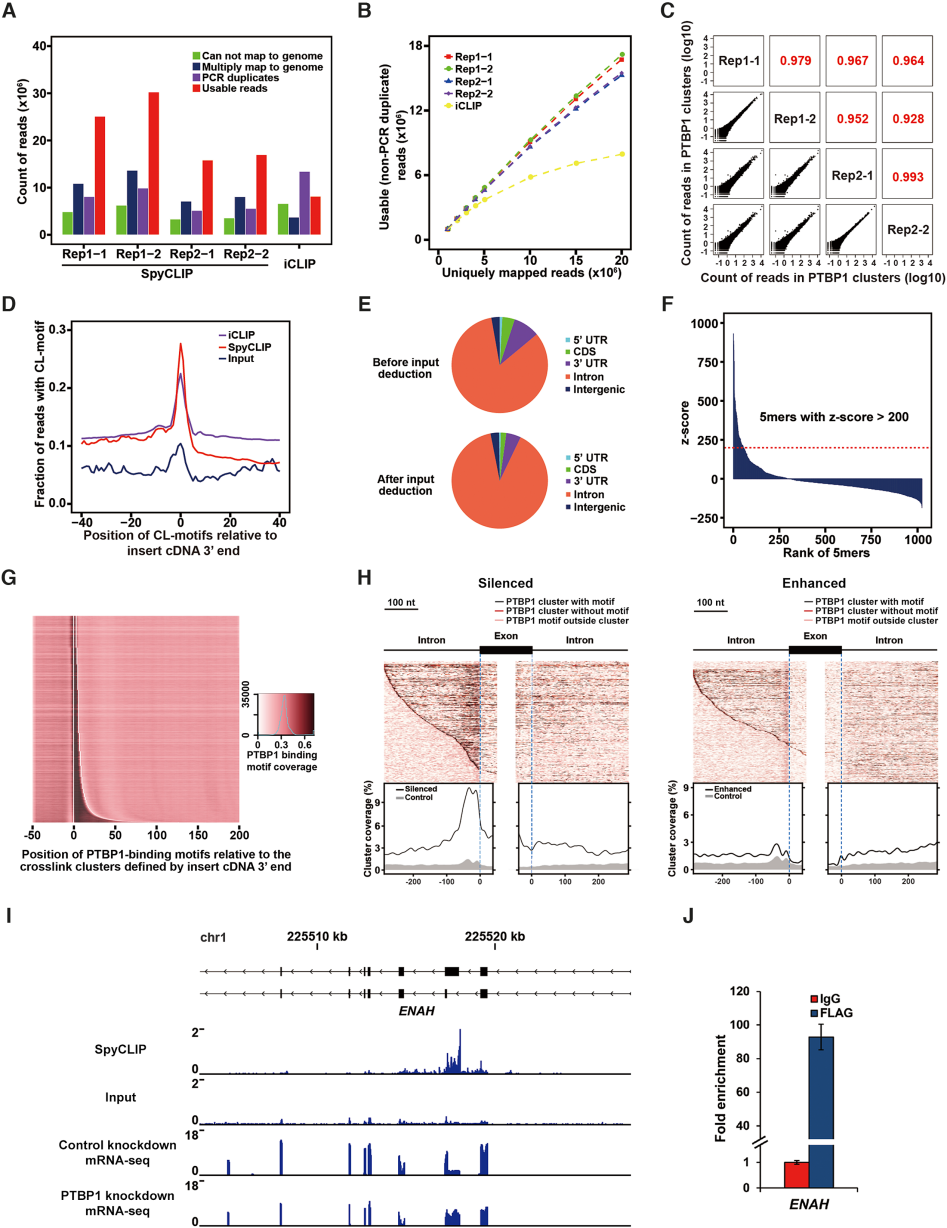
SpyCLIP generates high-quality binding maps for intronic RNA binding protein PTBP1. (**A**) Composition of sequencing reads from PTBP1 CLIP libraries using different methods. Usable reads refer to those that uniquely mapped to the genome after discarding PCR duplicates. (**B**) Saturation curves of non-PCR duplicated reads in different PTBP1 CLIP data. Random numbers of uniquely mapped reads were sampled successively for each experiment. (**C**) Reproducibility of PTBP1 SpyCLIP reads within the identified clusters. The first number within Rep1–1 (and so on) indicates a biological replicate, and the second number indicates a technical replicate. (**D**) The fraction of reads containing a CL motif at each site relative to the 3′ end of the insert cDNA. The CL motifs refer to pyrimidine-rich tetramers enriched at the site of UV crosslinking. (**E**) Distribution of PTBP1 SpyCLIP clusters in different genomic regions with or without input deduction. (**F**) The *z*-score of all different pentamers in PTBP1 SpyCLIP clusters. Pentamers with a *z*-score ≥200 are defined as PTBP1-binding motifs. (**G**) Heatmap showing the coverage of PTBP1-binding motifs in the PTBP1 SpyCLIP clusters. Each row shows the average coverage for 1200 clusters of similar length, sorted from the shortest to the longest clusters. The white line marks the start site and the median end of all clusters in the plot. (**H**) A binding map of PTBP1 near the intron-exon junction of regulated RNA in HEK293 cells. Each row of the heatmap shows a PTBP1-regulated exon with its flanking regions. The dark red line represents the position of clusters. The black and light red lines indicate the PTBP1 binding motifs inside or outside the clusters, respectively. The enrichment of clusters around the regulated exons compared to the control is plotted below. Left, silenced splicing sites; right, enhanced splicing sites. (**I**) Read density tracks of PTBP1 SpyCLIP data and PTBP1 knockdown RNA-seq data within the *ENAH* gene. The PTBP1 SpyCLIP identified clusters located exactly at the flanking region of the PTBP1-regulated exon. (**J**) qRT-PCR validation of PTBP1 binding sites within *ENAH*. The enrichment fold was calculated from the normalized *ENAH* mRNA levels from FLAG IP products versus IgG IP products. Error bars represent the standard deviation of three independent experiments.

The intronic binding site of RBP is one of the most technically challenging types to identify because of its low abundance. We therefore chose PTBP1 and RBFOX2, two well-characterized RBPs that primarily bind introns to regulate RNA processing, to evaluate the performance of SpyCLIP. We generated Lenti-X 293T cells stably expressing Spy-tagged RBPs at near-endogenous levels (Figure 1F and Supplementary Figure S1D) for SpyCLIP experiments and compared the resulting data with iCLIP (18) or eCLIP (11) data, which were also generated from HEK293 cells following exactly the same computational pipeline (19). For each RBP tested, two biological replicates were assessed, each containing two technical replicates. In addition, a universal input library (Supplementary Figure S2A and B) was prepared from parental cells for background subtraction. SpyCLIP produced highly reproducible sequencing results between technical and biological replicates (Figure 2C and Supplementary Figure S3C). More than 60% of the total reads could be uniquely mapped to the genome for both PTBP1 SpyCLIP and iCLIP libraries. Notably, only 16% of the total reads were PCR duplicates in all four PTBP1 SpyCLIP libraries, whereas 42% of the total reads in the iCLIP library represented PCR duplicates (Figure 2A and Supplementary Table S1). SpyCLIP, hence, generated a significantly higher percentage of usable sequences (uniquely mapped to the genome and non-PCR duplicates) than iCLIP did at the same sequencing depth (50% versus 25%) (Figure 2A). Similar fractions of uniquely mapped reads and PCR duplicates were observed in both SpyCLIP and eCLIP libraries for RBFOX2 (Supplementary Figure S3A and Supplementary Table S1). Moreover, no saturation was observed with SpyCLIP at near 20 million reads within the same range of uniquely mapped reads (Figure 2B and Supplementary Figure S3B).

UV crosslinked sites of RBPs enrich certain pyrimidine-rich tetramers, and such crosslink-associated (CL) motifs can be used to identify crosslink sites (18). PTBP1 is expected to show an especially high enrichment of the CL motifs due to its preference for binding to polypyrimidine sequences. Similar to iCLIP, SpyCLIP exhibited a strong enrichment of CL motifs over the corresponding input sample at the 3′ end of inserted cDNAs (Figure 2D), which resulted from the stalled reverse transcription by the crosslinked RBP, indicating SpyCLIP efficiently identifies RBP-RNA crosslink sites. We further analyzed the genomic distribution of SpyCLIP clusters and found that, for both PTBP1 and RBFOX2, the majority of SpyCLIP identified clusters are located in the intronic region. This pattern became even more obvious after input deduction (Figure 2E, Supplementary Figure S3F and Supplementary Table S2), which agrees with their intronic binding property. To determine the features of PTBP1 binding sites, we searched for pentamers enriched around the peaks of clusters (18) identified by PTBP1 SpyCLIP and discovered that 54 pentamers had an enrichment *z*-score ≥200 (Figure 2F). Consistent with the known UC-rich binding property of PTBP1 (20), all these pentamers are pyrimidine-rich (Supplementary Table S3) and thus were defined as PTBP1-binding motifs. We then examined the distribution of these PTBP1-binding motifs and found that they were highly overlapped with the PTBP1-specific SpyCLIP clusters (Figure 2G). By integrating our PTBP1 SpyCLIP data and published RNA-seq data of PTBP1 knockdown HEK293 cells (21), we drew an RNA splicing map to examine the distribution of clusters of assigned binding sites around silenced and enhanced exons and revealed that a substantial portion of SpyCLIP identified PTBP1 clusters were specifically enriched around the PTBP1 regulated exons (Figure 2H), suggesting that SpyCLIP identified clusters represent functional binding sites of PTBP1 (Figure 2I–J and Supplementary Figure S2D). Additionally, SpyCLIP faithfully recapitulated the known binding motif of RBFOX2 (Supplementary Figure S3H) and, when combining the RNA-seq data of RBFOX2 knockdown HEK293 cells (22), the RBFOX2 SpyCLIP identified clusters that were enriched around the RBFOX2-regulated exons (Supplementary Figure S3G), consistent with its known function in splicing regulation. Together, these observations demonstrate that SpyCLIP generates high-quality and reproducible libraries and identifies the authentic binding sites of RBPs with significantly reduced technical difficulty and procedure time.

### A single universal input control improves SpyCLIP specificity and sensitivity

**Figure 3.**
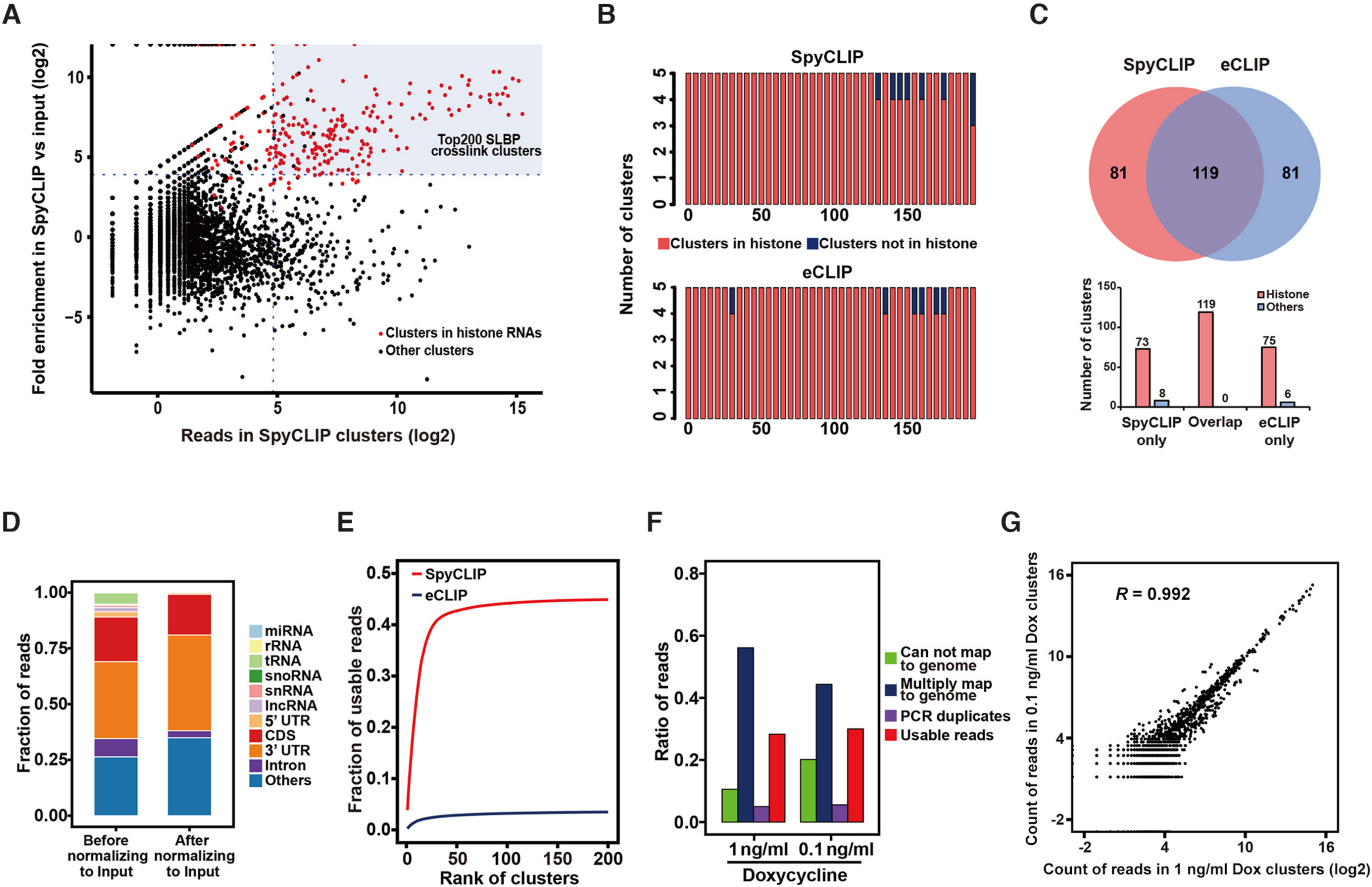
Improved SpyCLIP specificity and sensitivity after background deduction by a universal input library. (**A**) Fold enrichment for SLBP SpyCLIP clusters relative to the universal input library. The dots in red indicate the clusters overlapping with histone mRNA. The fold enrichment is defined as $\frac{{{\rm{number\ of\ reads\ in\ CLIP\ clusters}}}}{{{\rm{number\ of\ reads\ in\ input\ clusters}}}}$, and clusters with a fold enrichment > 15 are defined as SLBP-specific ones. (**B**) Histograms show the number of clusters located within histone mRNAs in the top 200 SLBP-specific clusters identified by SpyCLIP or eCLIP. The clusters were ranked and binned by their expression levels. (**C**) Overlap of SLBP-specific clusters identified by SpyCLIP and eCLIP. The number of clusters within histone mRNA or nonhistone RNAs in each section are shown in the lower panel. (**D**) Genomic distribution of SLBP SpyCLIP clusters before and after input deduction. Most of the noise signals removed by input normalization originated from abundant endogenous rRNAs, tRNAs, snRNAs and snoRNAs. (**E**) The cumulative fraction of usable reads in the top 200 SLBP-specific clusters identified by SpyCLIP or eCLIP. (**F**) Composition of sequencing reads from SLBP SpyCLIP libraries at different Dox induction concentrations. (**G**) Reproducibility of SLBP SpyCLIP reads within the identified clusters from libraries at different Dox induction concentrations.

Nonspecific background signals are commonly observed in CLIP sequencing data, which cause false-positive results and reduce mapping accuracy. The eCLIP method introduced a size-matched input control for background removal (11), which is effective but still depends on PAGE-membrane purification and needs to be independently prepared for each individual RBP and is therefore not suitable for high-throughput applications. We tested whether a common input library constructed from RNase I-treated total RNAs (Supplementary Figure S2A and B) could be used for general background removal. SLBP is a well-characterized RBP that exclusively binds to a highly conserved stem-loop structure in the 3′ UTR of histone mRNAs (23); therefore, the authentic binding sites can be easily distinguished from the background signals. Moreover, there are only approximately 120 known SLBP-bound RNAs in human cells, making it an ideal RBP candidate to evaluate the efficiency of the SpyCLIP method in characterizing RBPs with fewer bound RNAs. We performed peak calling for SLBP SpyCLIP and input libraries and calculated the enrichment ratio of each binding site by dividing the numbers of reads within CLIP and input overlapping peaks. A total of 3224 significantly enriched peaks were observed (Supplementary Tables S2 and S4), and most of the noise signals originated from abundant endogenous rRNA, tRNA, snRNA and snoRNA (Figure 3D). Notably, 96% of the top 200 and all top 100 SpyCLIP identified clusters (ranked by read numbers) overlapped with histone mRNAs, indicating the effectiveness of using the input library for noise deduction (Figure 3B). Among the 81 SpyCLIP-specific clusters, ∼90% were also in histone mRNAs, which is comparable to eCLIP (Figure 3C). Although the performance of SpyCLIP and eCLIP is seemingly similar, a much higher percentage of usable reads in SpyCLIP were mapped to the top 200 SLBP-specific clusters (Figure 3E), suggesting that SpyCLIP actually enriched more histone RNAs bound by SLBP than eCLIP did. Intriguingly, we found that one of the nonhistone RNAs bound by SLBP among the top 200 clusters, *PPP1R5B*, contains histone stem-loop-like structures (Supplementary Figure S4A), suggesting that SLBP might not exclusively bind to histone mRNA, which merits further investigation.

We routinely use 10^7^ cells induced with 1 ng/ml Dox for a single SpyCLIP experiment, consistent with eCLIP. However, we found that SpyCLIP using 10-fold fewer cells (10^6^ cells) (Supplementary Figure S3I–K) or a lower Dox concentration (0.1 ng/ml) (Figure 3F and G) for RBP induction still perfectly reproduced the results, as shown by RBFOX2 and SLBP. Moreover, a minimal correlation of reads was observed within SpyCLIP identified clusters between different RBPs, indicating highly unique clusters for each RBP (Supplementary Figure S5). In addition, the PTBP1 and RBFOX2 SpyCLIP libraries also generated a higher percentage of clusters located at intronic regions after deduction of the same input library (Figure 2E and Supplementary Figure S3F). Thus, we conclude that a single universal input library is sufficient to remove the majority of the noise and that SpyCLIP represents a cost-effective method with outstanding specificity and sensitivity.

### SpyCLIP reveals novel insights of miRNA target sites within the coding regions

**Figure 4.**
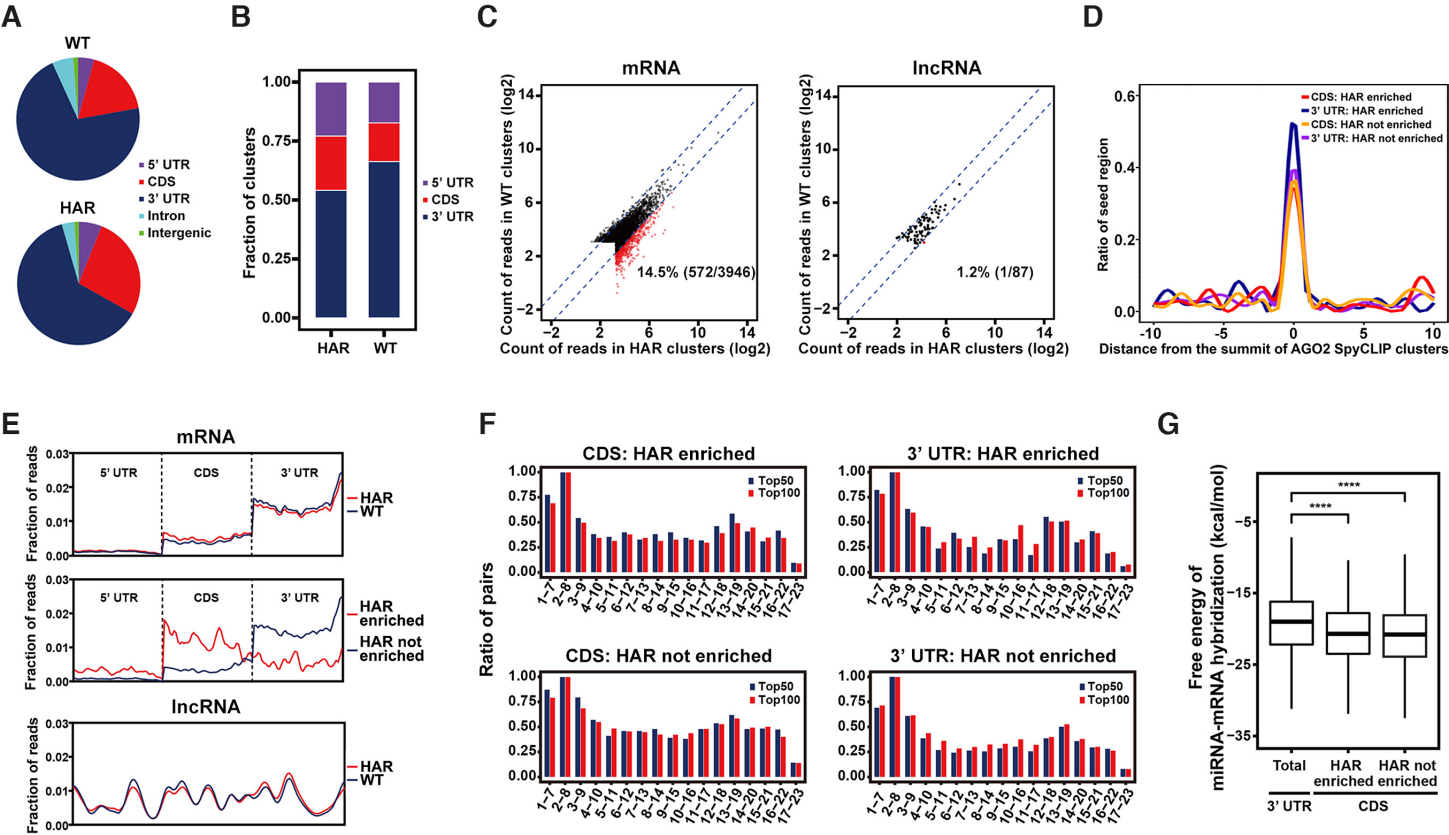
Characterization of miRNA target sites within the coding regions by SpyCLIP. (**A**) Distribution of AGO2 SpyCLIP clusters in different regions of protein-coding genes. WT: clusters identified under physiological conditions; HAR: clusters identified upon acute translation suppression by harringtonine. (**B**) Distribution of AGO2 SpyCLIP clusters in different genomic regions corrected by region size. WT and HAR are defined as in (A). (**C**) Changes of reads count in AGO2 SpyCLIP clusters between physiological (WT) and translation suppressed (HAR) conditions. Clusters in the HAR condition that exhibited at least a 2-fold increase over their counterparts in the WT condition are defined as HAR enriched and indicated by red dots. Other clusters are defined as HAR not enriched and indicated by black dots. (**D**) Complementary site density for all seed regions (nucleotides 2–8) of the top 100 miRNAs in the 20-nt region centered around the summit of AGO2 SpyCLIP clusters. (**E**) Distribution of reads within AGO2 SpyCLIP clusters along the transcripts of protein-coding or long noncoding RNAs. WT and HAR are defined as in (A). HAR enriched and HAR not enriched are defined as in (C). (**F**) Base-pairing tendencies of different regions within miRNAs to their complementary sites within AGO2 SpyCLIP identified clusters. Each 7-mer region (nucleotides 1–7, 2–8, 3–9, and so on) of the top 50 or top 100 AGO2-bound miRNAs was aligned with AGO2-bound clusters in the CDS or 3′ UTR of mRNAs identified by SpyCLIP to define a potential target site. The percentage of complementary sites in AGO2-bound clusters for each 7-mer region of the top 50 (blue bars) or top 100 (red bars) AGO2-bound miRNAs are plotted. (**H**) Free energy of miRNA-target duplex containing at least one 7-mer complement of the top 100 miRNAs defined in (F) in different categories of AGO2-bound clusters. ****P-value ≤ 0.0001.

MiRNAs are ∼21-nucleotide (nt) small noncoding RNAs that primarily bind to the 3′ UTR of protein-coding genes by imperfectly base-pairing with their target sequences (24). We applied SpyCLIP to the miRNA-binding protein AGO2 under normal or translation-repressed conditions. Consistent with previous CLIP studies, the AGO2 binding sites were primarily located within the 3′ UTRs of mRNAs and were less abundant in the CDSs and 5′ UTRs (25,26) (Figure 4A and B). When translation was blocked by the addition of the eukaryotic translation inhibitor HAR, the CDSs were more frequently targeted by AGO2 (Figure 4A–E). Moreover, existing CDS target sites that were weakly bound by AGO2 under physiological conditions were significantly enhanced (Figure. 4C–E). These observations suggest that the CDS contains many potential miRNA targets that are unmasked upon ribosome run-off, which is consistent with our previous study of the miRNA-mediated decay of nonsense mRNAs showing that the presence of premature stop codons allows the miRNA target sites in the downstream CDS to become functional (27). SpyCLIP also identified prevalent AGO2 binding along the full length of lncRNAs (Figure 4C–E), suggesting that miRNAs can also bind to lncRNAs. Whether lncRNAs contain ORFs that can be translated into functional peptides or proteins is a subject under debate (28,29). A few functional peptides encoded by lncRNAs underline the significance of their translation (30–33). Intriguingly, we found that the AGO2 binding pattern on the lncRNAs was evenly distributed and remained unchanged when translation was blocked by HAR, a result supporting the conclusion that functional ORF-containing lncRNAs are rare (28). Moreover, it is reasonable to expect that the AGO2-miRNA complex may participate in regulating lncRNA stability in the cytoplasm by recruiting the CCR4-NOT complex, as do mRNAs (34), which warrants future investigations.

We further investigated the base-pairing rules of 3′ UTR and CDS target sites. Predicted miRNA target sites were enriched at the summit of AGO2-specific clusters identified by SpyCLIP, suggesting a high confidence of these clusters (Figure 4d). The 3′ UTR miRNA target sites generally exhibited the highest base-pairing tendency within nucleotides 1–8 of the miRNAs, followed by nucleotides 12–19, regardless of whether they were constitutively present or enriched after translational repression (Figure 4F), which accurately corresponds to the previously defined ‘canonical’ and ‘3′ compensatory’ or ‘3′ supplementary’ target types, respectively (35), suggesting that SpyCLIP identifies authentic AGO2 binding sites in cells. Intriguingly, both categories of CDS target sites tended to display extra base-pairing in the middle and 3′ end regions of miRNAs when compared with their 3′ UTR counterparts (Figure 4F). We further calculated the base-pairing energy between the miRNA and its predicated mRNA target and found that CDS target sites indeed exhibited stronger miRNA-mRNA target interactions than 3′ UTR targets (Figure 4G). These observations are not unexpected because the AGO2-miRNA complex may require extra base-pairing to compete with the translating ribosomes to remain bound with the CDS target sites. We propose that not merely the base-paring energies but the base-pairing patterns between miRNAs and their targets in the 3′ UTRs are optimized to fit or induce the conformational changes in the RNA-induced silencing complex (RISC) for efficient gene silencing, while miRNA targets in the CDSs have not been highly selected during evolution and their bindings with miRNAs rely heavily on thermodynamic stability, which may explain the requirement for more extensive base-pairing for such targets. Together, these results indicate that SpyCLIP generates high-quality data that not only faithfully reveal the known properties of the AGO2-miRNA complex but also provide mechanistic insight to better understand the CDS miRNA targets, which remain to be thoroughly studied.

## DISCUSSION

In this study, by blending the SpyTag-SpyCatcher chemistry and the CLIP methodology, we have developed an easy-to-use and high-throughput compatible SpyCLIP platform that features a stringent covalent linkage-based RNP purification scheme and a robust library construction strategy. The covalent interaction between SpyTag and SpyCatcher overcomes the limitations of the equilibrium-based antigen-antibody system and allows extensive washes under harsh conditions, which completely circumvents the PAGE-membrane purification steps required by conventional CLIP methods and produces highly complex and reproducible libraries with outstanding specificity and sensitivity. The SpyCatcher beads are able to directly enrich Spy-tagged RBPs in crude cell lysate; however, the efficiency is significantly lower than that of FLAG-purified products (∼10% versus >99%, data not shown), probably because of the slower kinetics for covalent linkage formation than antibody–antigen recognition and the high complexity of the proteins present in the total cell lysate. To ensure maximal SpyTag-SpyCatcher covalent linkage formation and RBP recovery, we routinely perform FLAG IP first. It is reasonable to expect that, for some abundant RNPs, a single round of SpyCatcher pull-down might be sufficient to provide satisfactory results, which could further simplify the procedure of the current protocol. Alternatively, immunoprecipitation using SpyTag-specific antibodies can be performed first to enrich Spy-tagged RBPs before they are covalently linked to SpyCatcher beads under the circumstances that the inserted tag size needs to be maintained at a minimum. We have also carefully designed the library construction procedure such that all of the reactions can be continuously performed on beads or in solution, which dramatically reduces the technical difficulty, procedure time and cost of the protocol. In addition, when normalized against a universal input control, the signal-to-noise ratio of SpyCLIP data is further improved. The SpyCLIP protocol is able to generate sequencing-ready libraries within only three days without any SDS-PAGE separation and membrane transfer of RNP, labeling of RNA for visualization or RNA precipitation steps, representing the most user-friendly and cost-effective CLIP platform so far. Using this new tool, we generated highly complex and reproducible RNA binding maps with outstanding sensitivity and specificity for multiple well-studied RBPs and revealed novel insights into their functional mechanisms.

Currently, all commonly used CLIP method varieties, such as iCLIP, eCLIP and irCLIP, require labor-intensive gel purification steps. SpyCLIP completely omits RBP-RNA gel purification steps and provides highly specific and sensitive RNA binding maps with a quality comparable to that of iCLIP and eCLIP. The main goal for the development of SpyCLIP is to provide a user-friendly and high-throughput compatible tool with robust performance for systematic studies of RBPs; therefore, we did not intensively optimize the protocol for using less input cells than the standard protocols of other CLIP methods. Although irCLIP, which relies on nonradioactive labeling of RNA for visualization and gel purification, was reported to use less starting material, SpyCLIP achieves better reproducibility than irCLIP at the cellular input level of 10^6^ cells (Supplementary Figure 3J and K), and it is reasonable to expect that the minimal input level could be further reduced by further optimization of the SpyCLIP protocol. During the submission of our study, a covalent linkage-based CLIP method, GoldCLIP, was reported to apply denaturing washing conditions during RNP purification; however, the fused HaloTag is larger than 30 kDa. In contrast, the 13-aa size of SpyTag not only has a marginal effect on protein activity but is also superior for generating knock-in animals for *in vivo* studies. Therefore, SpyTag-SpyCatcher chemistry confers unique advantages to the SpyCLIP method, making it a highly specific, easy-to-use and high-throughput compatible platform for systematic characterization of protein–RNA interactions.

## DATA AVAILABILITY

Raw sequencing data of PTBP1, RBFOX2, SLBP, AGO2 and input SpyCLIP libraries generated from Lenti-X 293T cells were deposited at the Gene Expression Omnibus (GSE114720).

## Supplementary Material

Supplementary Data
