# Supplementary material for: SpyCLIP: an easy-to-use and high-throughput compatible CLIP platform for the characterization of protein–RNA interactions with high accuracy
Source: Nucleic Acids Res. 2019 Jan 31;47(6):e33. doi: 10.1093/nar/gkz049 (PMC6451120; doi:10.1093/nar/gkz049)
Supplement: Supplementary Data [file gkz049_supplemental_files.zip › Supplementary Figures and Protocol.pdf]

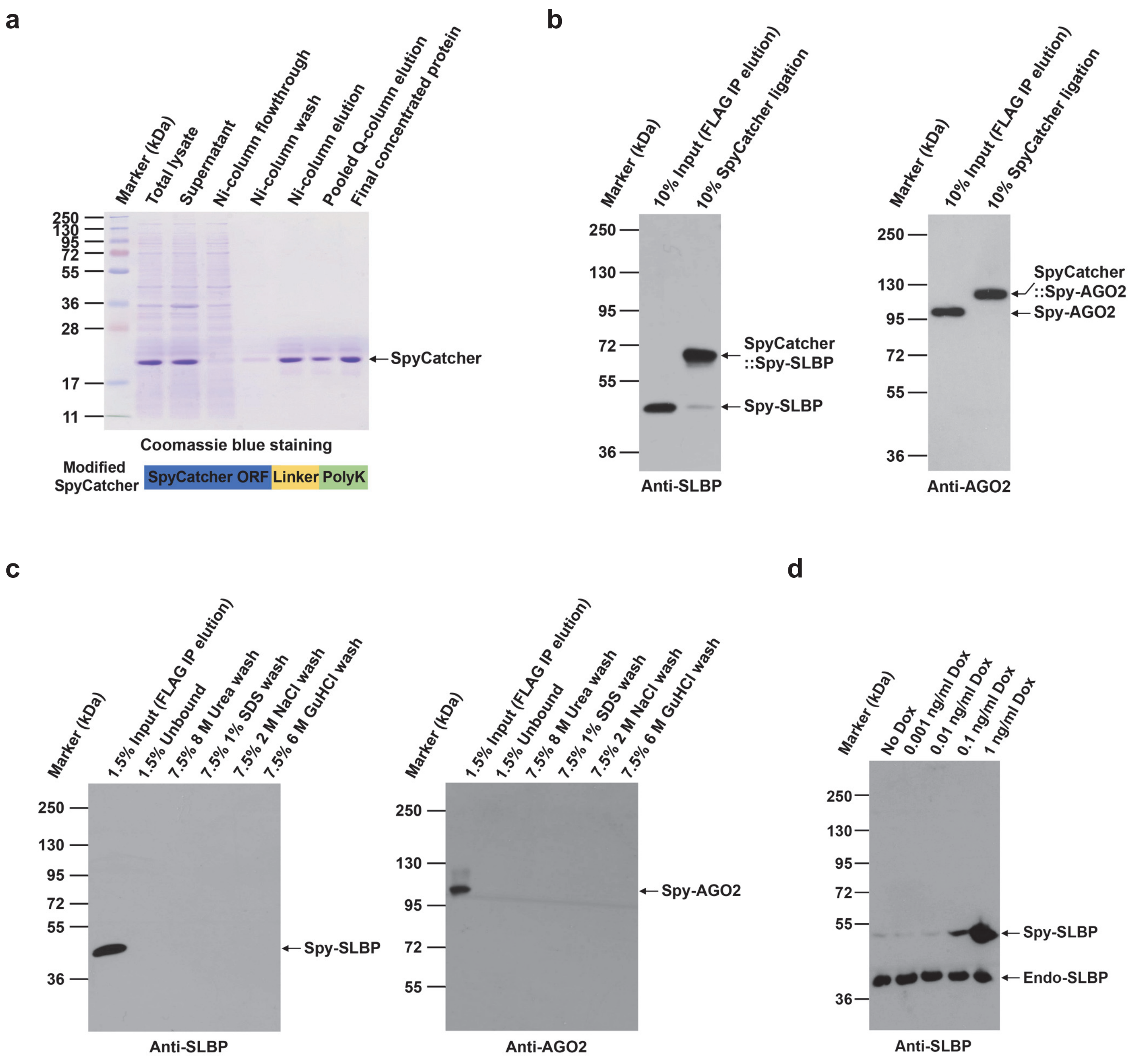

Supplementary Figure 1. The SpyTag-SpyCatcher pull-down system

(**a**) High-quality SpyCatcher proteins were purified via a nickel column followed by an anion-exchange column. Eight lysine residues were added to the C-terminus of the original SpyCatcher ORF, providing extra free amino groups for site-directed coupling to the Epoxy beads. (**b**) The SpyCatcher protein efficiently formed a stable complex with Spy-tagged SLBP or AGO2 proteins in SpyCLIP lysis buffer. FLAG- and Spy-tagged SLBP or AGO2 proteins from 5×10^6 Lenti-X 293T stable cells were enriched from cell lysates by anti-FLAG beads and eluted by PreScission Protease cleavage. The eluted proteins were incubated with 10 μg of purified SpyCatcher proteins for 1 hour at 25 °C to form covalent linkages. (**c**) The covalent SpyTag-SpyCatcher pull-down system can withstand harsh washing conditions, including buffers containing 8 M urea, 1% SDS, 2 M NaCl and 6 M guanidine hydrochloride. FLAG- and Spy-tagged SLBP or AGO2 proteins from 1×10^7 Lenti-X 293T stable cells were enriched from cell lysates by anti-FLAG beads and eluted by PreScission Protease cleavage. The eluted protein was then subjected to SpyCatcher pull-down and sequential washing steps as indicated. (**d**) Comparison of the expression levels of dox-induced Spy-tagged SLBP with its endogenous counterpart in Lenti-X 293T cells.

Supplementary Figure 2

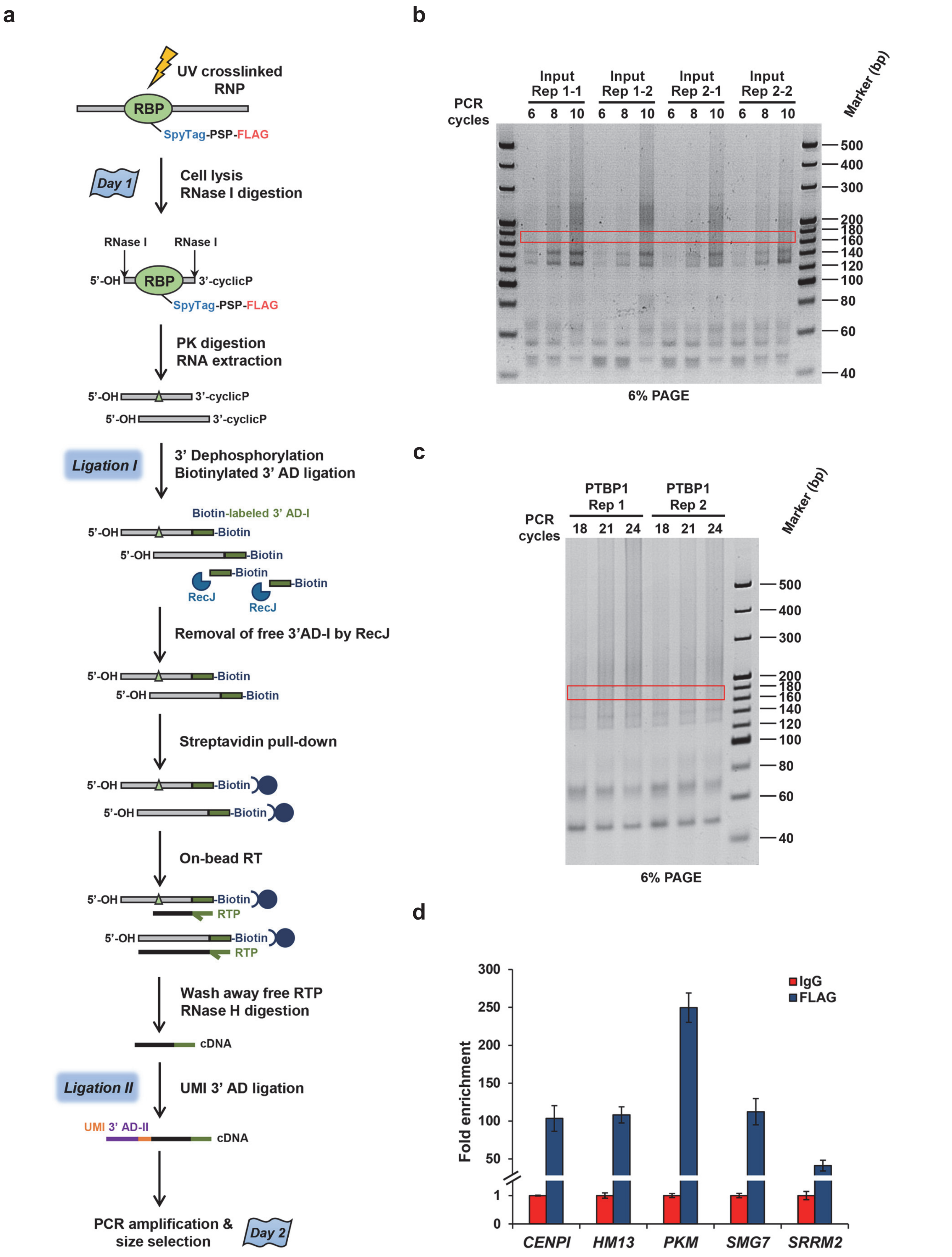

Supplementary Figure 2. Schematic representation of the input library preparation procedure for SpyCLIP and validation of PTBP1 SpyCLIP targets

(**a**) UV crosslinked cells were lysed and partially digested with RNase I. The lysate was treated with proteinase K, and RNAs were extracted. The RNA fragments were dephosphorylated by PNK and ligated with a biotin-labeled 3' adapter. Free adapters were removed by digestion with RecJ. Ligated RNA fragments were captured by streptavidin beads, and the following steps were the same as SpyCLIP library construction. (**b**) Amplification of the input library constructed from parental Lenti-X 293T cells. The region marked by the rectangle (160-180 bp) was recovered for deep sequencing. The first number within Rep 1-1 (and so on) indicates a biological replicate, and the second number indicates a technical replicate. (**c**) Amplification of the PTBP1 SpyCLIP library by different PCR circles. The region marked by the rectangle (160-180 bp) was recovered for deep sequencing. (**d**) qRT-PCR validation of PTBP1 binding sites within five randomly selected genes. The enrichment fold was calculated from the normalized target mRNA levels from FLAG IP products versus IgG IP products. Error bars represent the standard deviation of three independent experiments.

Supplementary Figure 3

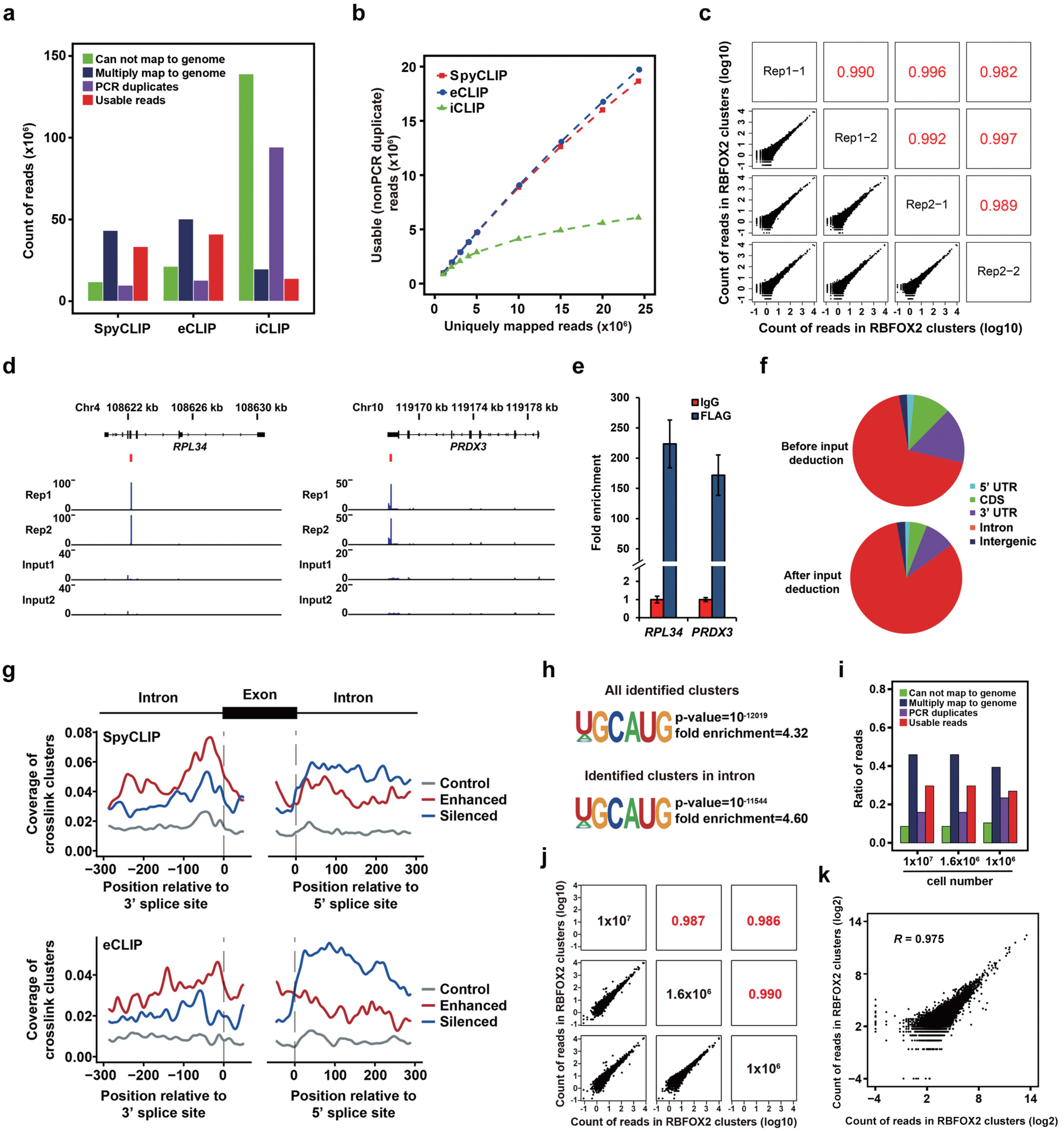

Supplementary Figure 3. SpyCLIP identifies authentic RBFOX2 binding sites

(**a**) Composition of sequencing reads from RBFOX2 CLIP libraries using different methods. Usable reads refer to those that uniquely mapped to the genome after discarding PCR duplicates. (**b**) Saturation curves of non-PCR duplicated reads in different RBFOX2 CLIP data. Random numbers of uniquely mapped reads were sampled successively for each experiment. (**c**) Reproducibility of RBFOX2 SpyCLIP reads within the identified clusters. The first number within Rep1-1 (and so on) indicates a biological replicate, and the second number indicates a technical replicate. (**d**) Read distribution of the identified clusters within *RPL34* and *PRDX3* genes in RBFOX2 SpyCLIP and input samples. The red line indicates the location of the UGCAUGCA binding motif for RBFOX2. (**e**) The qRT-PCR validation of RBFOX2 binding sites within *RPL34* and *PRDX3* genes shown in (**d**). The enrichment fold was calculated from the normalized *RPL34* or *PRDX3* mRNA levels from FLAG IP products versus IgG IP products. Error bars represent the standard deviation of three independent experiments. (**f**) Location of RBFOX2 SpyCLIP clusters in different regions of protein-coding genes with or without input deduction. (**g**) A binding site map of RBFOX2 near the intron-exon junction of regulated RNA in HEK293 cells. The enrichment of SpyCLIP or eCLIP identified clusters near the regulated exons compared to the control is plotted. (**h**) Sequence logos corresponding to enriched sequence elements identified by *de novo* motif analysis of RBFOX2 SpyCLIP clusters. (**i**) Composition of sequencing reads from RBFOX2 SpyCLIP libraries of different input cell numbers. (**j**) Reproducibility of RBFOX2 SpyCLIP reads within the identified clusters from libraries of different input cell numbers. (**k**) Reproducibility of RBFOX2 SpyCLIP reads within the identified clusters from libraries generated from two independent experiments using $1×10^6$ cells.

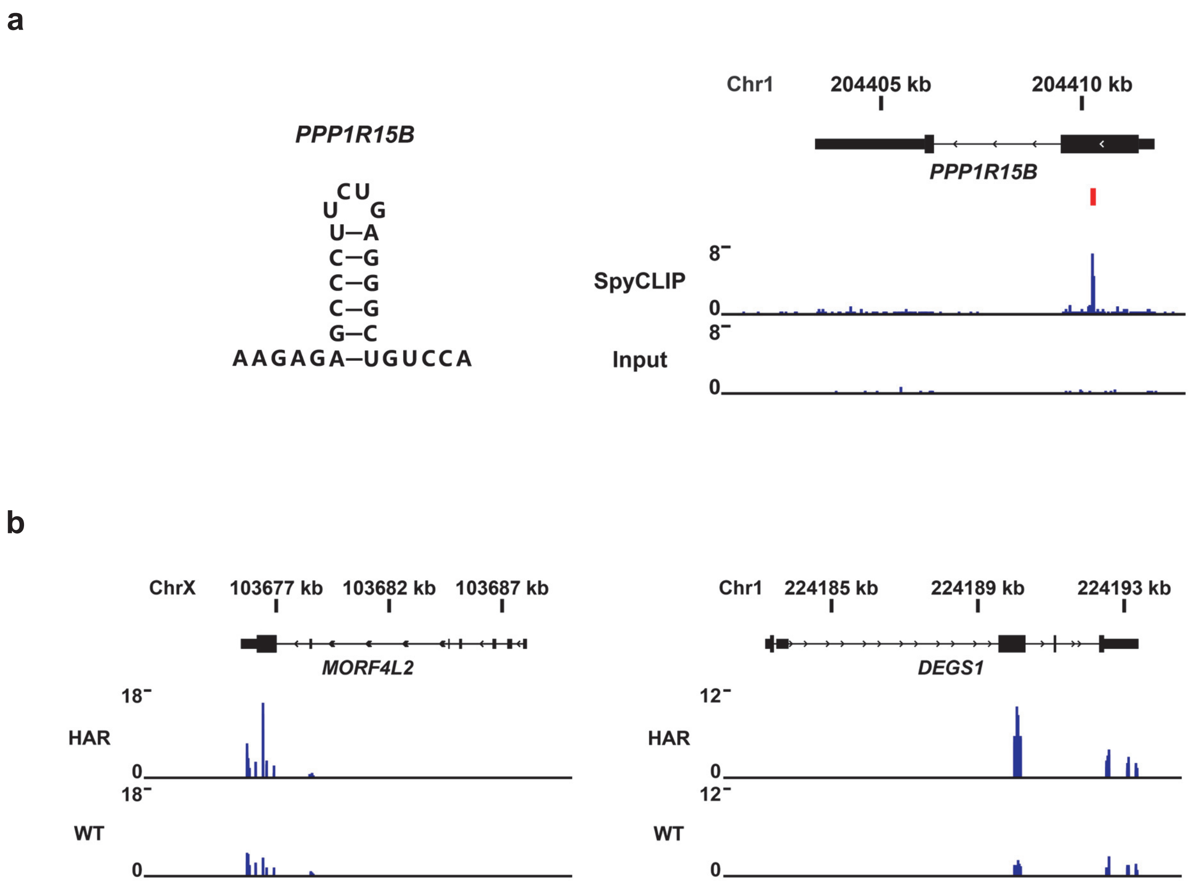

Supplementary Figure 4. Examples of SLBP and AGO2 SpyCLIP targets

(**a**) The read distribution of the identified clusters within the *PPP1R15B* gene in SLBP SpyCLIP libraries. *PPP1R15B* is a nonhistone gene that harbors a histone stem-loop-like structure and is specifically identified by SpyCLIP. The location of the stem-loop structure is indicated in red. (**b**) The read distribution of the identified clusters within *MORF4L2* (left) and *DEGS1* (right) genes in AGO2 SpyCLIP libraries. For both genes, several target sites within the CDS exhibited higher AGO2 binding upon translation inhibition by HAR, while target sites within the 3' UTR did not change significantly.

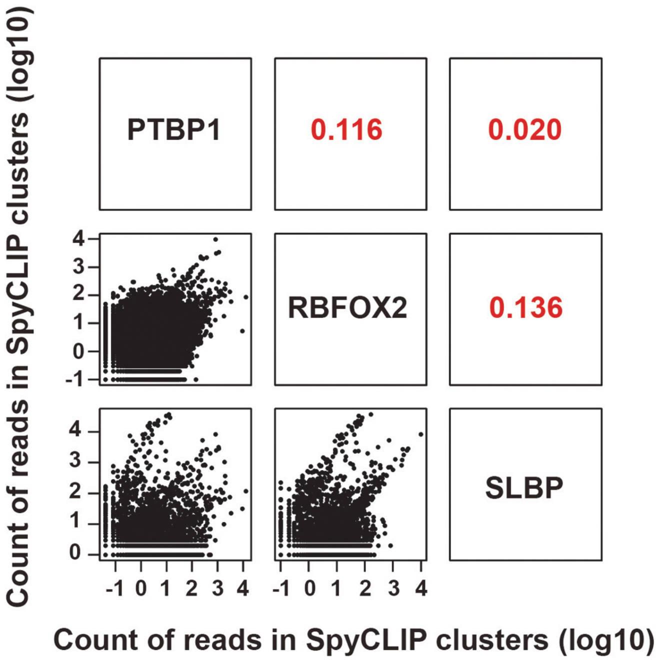

Supplementary Figure 5. Reproducibility of SpyCLIP reads within the identified clusters of three different RBPs

Pearson correlations of the SpyCLIP reads within the clusters identified in the SpyCLIP libraries for PTBP1, RBFOX2 and SLBP. Low R values indicate highly unique clusters for each RBP.

Supplementary protocol 1: SpyCLIP Procedures

Preparation of cell pellets (<u>Any time before a SpyCLIP experiment)</u>

Buffer:

PBS (HyClone, SH30256.01)

Harvest cells at 80% confluency from two 10-cm dishes for each CLIP experiment as follows:

1. Aspirate the culture medium and wash cells with 7 ml of cold PBS.
2. Aspirate the PBS and add another 7 ml of fresh cold PBS.
3. Place the dishes on a plate containing ice-water mixture and irradiate the cells at 400 mJ/cm^2 in a UV Crosslinker (UVP, CL-1000).
4. Scrape the cells and transfer them to a 15-ml conical tube.
5. Pellet the cells by centrifugation at 800 g for 3 min.
6. Aspirate the supernatant, resuspend the cell pellet in 1 ml of cold PBS and transfer it to a 1.5-ml microcentrifuge tube.
7. Pellet the cells by centrifugation at 800 g for 3 min.
8. Aspirate the supernatant and freeze the cell pellet at -80 °C until use.

Day 1 (Start SpyCLIP in the afternoon)

FLAG IP

Buffer:

Lysis buffer: 50 mM Tris (pH 7.4), 150 mM NaCl, 0.5% sodium deoxycholate, 0.1% SDS, 1% Triton X-100, add 1:100 protease inhibitor cocktail (Sigma-Aldrich, P8340) freshly

1. Retrieve a cell pellet from -80 °C, resuspend the pellet with 1 ml of Lysis buffer and incubate it on ice for 10 min.
2. Centrifuge at 14000 g for 10 min and transfer the supernatant to a new 1.5-ml microcentrifuge tube containing 2 µl of Turbo DNase (2 U/µl, Invitrogen, AM2238).

We use 1.5-ml LoBind microcentrifuge tubes (Eppendorf, 0030108051) throughout the whole process of SpyCLIP.

3. Make a 1:200 dilution of RNase I: 99.5 µl PBS + 0.5 µl RNase I (100 U/µl, Invitrogen, AM2295).
4. Add 10 µl of diluted RNase I to the lysate, immediately incubate in a thermomixer at 1100 rpm, 37 °C for 5 min exactly, and then transfer to ice.
5. Add 100 µl of pre-washed anti-FLAG magnetic beads (15 mg/ml, MBL, M185-11) to the lysate and rotate the mixture at 25 °C for 40 min.
6. Collect the beads with a magnetic stand and discard the supernatant.
7. Wash the beads with 1 ml of Lysis buffer for 3× 5 min.

Removal of 2', 3' cyclic monophosphate at the 3' end of RNase I-treated RNAs

Buffer:

PNK wash buffer: 20 mM Tris (pH 7.4), 10 mM $MgCl_2$, 0.2% Tween 20

1. Wash the beads twice with 1 ml of PNK wash buffer.
2. Prepare PNK master mix:

DEPC-treated H_2O	24.25 µl
10× PNK buffer	3 µl
PNK (10 U/µl, NEB, M0201S)	1 µl
RiboLock (40 U/µl, Thermo Scientific, EO0381)	0.75 µl
Turbo DNase (2 U/µl, Invitrogen, AM2238)	1 µl
**Total**	**30 µl**

3. Resuspend the beads with 30 µl of PNK mix and rotate the mixture at 37 °C for 20 min.
4. Collect the beads with a magnetic stand and discard the supernatant.
5. Wash the beads with 1 ml of Lysis buffer for 3× 5 min to completely remove the DNase in the PNK mix.
6. Wash the beads twice with 1 ml of PNK wash buffer.

Ligation I: Biotinylated 3' adapter ligation

1. Prepare Ligation I master mix:

DEPC-treated H_2O	17.5 µl
10× T4 RNA ligase buffer	3 µl
BSA (2 mg/ml)	3 µl
Biotinylated 3' adapter (20 µM)	2.5 µl
RiboLock (40 U/µl)	1 µl
T4 RNA Ligase 1 (30 U/µl, NEB, M0437M)	3 µl
**Total**	**30 µl**

2. Resuspend the beads with 30 µl of Ligation I mix and rotate the mixture at 16 °C for 12 hours.

Day 2 (Morning)

Release RNP from FLAG beads

Buffers:

Lysis buffer: 50 mM Tris (pH 7.4), 150 mM NaCl, 0.5% sodium deoxycholate, 0.1% SDS, 1% Triton X-100

PSP buffer: 50 mM Tris (pH 7.4), 150 mM NaCl, 0.1% Triton X-100, 1 mM EDTA, 1 mM DTT

1. Collect the beads with a magnetic stand and discard the supernatant.
2. Wash the beads with 1 ml of Lysis buffer for 3× 5 min.
3. Wash the beads twice with 1 ml of PSP buffer.
4. Prepare Elution I master mix:

PSP buffer	48 µl
PSP (2 U/µl, GE Healthcare, 27-0843-01)	1 µl
Protease Inhibitor Cocktail (Sigma-Aldrich, P8340)	0.5 µl
RiboLock (40 U/µl)	0.5 µl

**Total**	**50 µl**

5. Resuspend the beads with 50 µl of Elution I mix and incubate the reaction in thermomixer at 22 °C for 2 hours with shaking at 1100 rpm every 15 sec.
6. Collect the beads with a magnetic stand, transfer the supernatant to a new 1.5-ml tube, and then store on ice.
7. Prepare Elution II master mix:

Lysis buffer	49 µl
Protease Inhibitor Cocktail	0.5 µl
RiboLock (40 U/µl)	0.5 µl
**Total**	**50 µl**

8. Resuspend the beads with 50 µl of Elution II mix and incubate the reaction in a thermomixer at 22 °C for 20 min with shaking at 1100 rpm every 15 sec.
9. Collect the beads with a magnetic stand and combine Elution II with Elution I.

SpyCatcher pull-down

Buffer:

Spy pull-down buffer: 50 mM Tris (pH 7.4), 150 mM NaCl, 1% Triton X-100

1. Dilute the elution mixture with 350 µl Spy pull-down buffer and add 4 µl protease inhibitor cocktail and 1.5 µl RiboLock.
2. Add 50 µl of pre-washed SpyCatcher beads (see **Online Methods**) to the elution mixture and rotate at 25 °C for 1 hour.

Day 2 (Afternoon)

Stringent washes

Buffers:

Urea wash buffer: 100 mM Tris (pH 7.4), 150 mM NaCl, 8 M urea, 0.2% Triton X-100

SDS wash buffer: 100 mM Tris (pH 7.4), 150 mM NaCl, 1% SDS, 0.2% Triton X-100

High-salt wash buffer: 50 mM Tris (pH 7.4), 2 M NaCl, 0.2% Triton X-100

Low-salt wash buffer: 50 mM Tris (pH 7.4), 0.2% Triton X-100

Spy pull-down buffer: 50 mM Tris (pH 7.4), 150 mM NaCl, 1% Triton X-100

1. Collect the beads with a magnetic stand and discard the supernatant.
2. Wash the beads with 1 ml of Urea wash buffer for 2× 8 min.
3. Wash the beads once with 1 ml of Spy pull-down buffer.
4. Wash the beads with 1 ml of SDS wash buffer for 2× 8 min.
5. Wash the beads once with 1 ml of Spy pull-down buffer.
6. Wash the beads with 1 ml of High-salt wash buffer for 2× 8 min.
7. Wash the beads with 1 ml of Low-salt wash buffer for 2× 8 min.

Release RNA from SpyCatcher beads

Buffer:

PK buffer: 50 mM Tris (pH 7.4), 50 mM NaCl, 1 mM EDTA, 0.2% SDS

1. Prepare PK digestion master mix:

PK buffer	130 µl
Proteinase K (20 mg/ml, Roche, 3115828001)	20 µl
**Total**	**150 µl**

2. Collect the beads with a magnetic stand and discard the Low-salt wash buffer.
3. Resuspend the beads with 150 µl of PK digestion mix.
4. Incubate the reaction in thermomixer at 50 °C for 40 min with shaking at 1100 rpm every 15 sec.

RNA recovery by Streptavidin beads

Buffers:

Streptavidin pull-down buffer: 10 mM Tris (pH 7.4), 1 M NaCl, 1 mM EDTA, 0.2% Tween 20

Streptavidin wash buffer: 10 mM Tris (pH 7.4), 0.2% Tween 20

1. Collect the beads with a magnetic stand and transfer the supernatant to a new 1.5-ml tube.
2. Add 0.3 mg of pre-washed Streptavidin beads (10 mg/ml, BioMag, BMH1000-1) resuspended in 150 µl of Streptavidin pull-down buffer to the PK digestion mixture.
3. Rotate the mixture at 25 °C for 20 min.
4. Wash the beads with 1 ml of Streptavidin pull-down buffer for 3× 5 min.
5. Wash the beads twice with 1 ml of Streptavidin wash buffer.

On-bead reverse transcription

Buffers:

Streptavidin pull-down buffer: 10 mM Tris (pH 7.4), 1 M NaCl, 1 mM EDTA, 0.2% Tween 20

Streptavidin wash buffer: 10 mM Tris (pH 7.4), 0.2% Tween 20

1. Prepare RT master mix:

DEPC-treated H_2O	10.5 µl
RT primer (50 µM)	1 µl
dNTP mix (10 mM each)	2 µl
Incubate at 65 °C for 5 min, immediately chill on ice, and then add the following:	
5× Reaction Buffer	4 µl
BeyoRT II M-MLV (200 U/µl, Beyotime, D7161)	2 µl
RiboLock (40 U/µl)	0.5 µl
**Total**	**20 µl**

2. Resuspend the washed beads with 20 µl of RT mix and rotate the mixture at 44 °C for 1 hour.
3. Adjust the temperature to 37 °C and wash the beads with 1 ml of Streptavidin pull-down buffer for 3× 5 min.

4. Wash the beads twice with 1 ml of Streptavidin wash buffer.

Release cDNA into solution

1. Prepare RNase H digestion master mix:

DEPC-treated H_2O	8.5 µl
10× RNase H buffer	1 µl
RNase H (5 U/µl, NEB, M0297S)	0.5 µl
**Total**	**10 µl**

2. Collect the beads with a magnetic stand and discard the Streptavidin wash buffer.
3. Resuspend the beads with 10 µl of RNase H mix and rotate the mixture at 37 °C for 20 min.
4. Incubate the reaction at 85 °C for 5 min in thermomixer with shaking every 15 sec.
5. Immediately cool the tube on ice for at least 1 min.
6. Spin briefly and transfer 9.2 µl supernatant to a new 1.5-ml tube.

Ligation II: UMI 3' adapter ligation

1. Prepare Ligation II master mix:

UMI 3' adapter (100 µM)	0.5 µl
DMSO (100%)	2 µl
10× T4 RNA ligase buffer	3 µl
ATP (100 mM)	0.3 µl
50% PEG 8000 (wt/vol))	12 µl
T4 RNA Ligase 1 (30 U/µl)	3 µl
**Total**	**20.8 µl**

2. Add the Ligation II mix to the 9.2 µl cDNA solution and vortex briefly.
3. Incubate the reaction in thermomixer at 25 °C for 16 hours, shaking it for 15 sec every 2 min.

The cDNA library can be stored at -80 °C or directly used for subsequent amplification and size-selection.

PCR amplification of the cDNA library **(Any time before deep sequencing)**

1. Prepare PCR master mix:

Ligated cDNA	13.2 µl
RP1 primer (10 µM)	10.56 µl
RPI primer (10 µM)	10.56 µl
10× KOD buffer	26.4 µl
dNTP (2.5 mM each)	26.4 µl
Mg_2SO_4 (50 mM)	15.84 µl
KOD-Plus-Neo (1 U/µl, Toyobo, KOD-401)	6.6 µl
H_2O	154.44 µl
**Total**	**264 µl**

2. Aliquot 80 µl of PCR mixture into three 0.2-ml PCR tubes.
3. Program the PCR as follows: denaturation at 95 °C for 3 min (1 cycle); 6 cycles of a three-step program of 98 °C for 10 sec, 60 °C for 15 sec and 68 °C for 15 sec; 14 cycles of a two-step program of 98 °C for 10 sec, 68 °C for 30 sec.
4. Combine the PCR products from all three tubes and purify them using a DNA purification kit (TIANGEN, DP214).
5. Elute the purified PCR product with 30 µl of 10 mM Tris (pH 7.4).

Size-selection of the cDNA library

Buffers:

5× TBE: 445 mM Tris, 445 mM boric acid, 10 mM EDTA

TE buffer: 10 mM Tirs (pH 7.4), 1 mM EDTA

1. Pour a 6% polyacrylamide gel by mixing 10 ml of 30% gel stock solution, 10 ml of 5×

TBE buffer, 30 ml of water, 500 μl of 10% APS and 50 μl of TEMED.

2. Add 6 μl of 6× Loading Buffer (Takara, 9156) to the 30 μl purified PCR solution, and load 30 μl into one well. In addition, load a 20-bp DNA Ladder (Takara, 3420A) on the side of the gel.
3. Run the gel in 1× TBE buffer at 160 V until the bromophenol blue dye is 11 cm away from the bottom of the well.
4. Stain the gel with Gel-Green (Beyotime, D0143) in 1× TBE for 20 min with gentle shaking at room temperature.
5. Visualize the DNA using a blue light transilluminator (TIANGEN, OSE-470).
6. Excise the region between 160-180 bp with a clean razor blade.
7. Use a hot needle to create a hole in the bottom of a 0.6-ml microcentrifuge tube, and place the gel slice into it.
8. Place the 0.6-ml microcentrifuge tube into a 2-ml microcentrifuge tube and centrifuge at 14000 g for 1 minute.
9. Add 400 μl of TE buffer to the crushed gel and rotate at 25 °C overnight.
10. Transfer the gel mixture to a Spin-X column (Corning, CLS8160) and centrifuge at 14000 g for 1 min.
11. Discard the gel debris and add 40 μl of 3 M sodium acetate, pH 5.5 (Invitrogen, AM9740), 800 μl of 100% ethanol and 2 μl of linear acrylamide (5 mg/ml, BBI, A610548-0001) to the elution and vortex briefly.
12. Precipitate the DNA library for 2 hours to overnight at -20 °C.
13. Pellet the DNA by centrifugation at 14000 g for 15 min at 4 °C.
14. Wash the DNA pellet once with 1 ml of 70% ethanol.
15. Discard the ethanol and allow the pellet to air-dry.
16. Dissolve the pellet in the appropriate amount of water.
17. The library solution is now ready for quantification using a Qubit Fluorometer (Invitrogen, Q32866) and deep sequencing.

Supplementary protocol 2: SpyCLIP Input Procedures

Prepare cell pellets as described for SpyCLIP.

Note 1. For most constitutively expressed RBPs that are induced at near physiological levels, construct one universal input library from parental cells that are used to establish Spy-Tagged protein-expressing stable cell lines.

Note 2. For RBPs that are induced ectopically, or when any stimulations are applied to the cells, construct individual input libraries from the same cells used for construction of SpyCLIP libraries. Aliquot 10% of the total lysate after RNase I treatment for RNA extraction and subsequent library construction steps.

Day 1

RNA processing before ligation

Buffer:

Lysis buffer: 50 mM Tris (pH 7.4), 150 mM NaCl, 0.5% sodium deoxycholate, 0.1% SDS, 1% Triton X-100, add 1:100 protease inhibitor cocktail freshly

1. Retrieve a cell pellet from -80 °C, resuspend the pellet with 1 ml of Lysis buffer and incubate it on ice for 10 min.
2. Centrifuge at 14000 g for 10 min and transfer the supernatant to a new 1.5-ml microcentrifuge tube containing 2 µl Turbo DNase. We use 1.5-ml LoBind microcentrifuge tubes throughout the whole process of Input library preparation.
3. Make a 1:200 dilution of RNase I: 99.5 µl PBS + 0.5 µl RNase I (100 U/µl).
4. Add 10 µl of diluted RNase I to the lysate, immediately incubate in a thermomixer at 1100 rpm, 37 °C for 5 min exactly, and then transfer it to ice.
5. Transfer 100 µl of the lysate to a new 1.5-ml tube and adjust to PK buffer by adding 2

µl of 10% SDS and 2 µl of 50 mM EDTA.

6. Add 10 µl of Proteinase K (20 mg/ml) to the lysate and incubate it in a thermomixer at 50 °C for 40 min with shaking every 15 sec.
7. Extract RNA using TRIzol LS reagent (Invitrogen, 10296028) and dissolve the RNA pellets in 15 µl of DEPC-treated water.

Removal of 2', 3' cyclic monophosphate at the 3' end of the RNase I-treated RNAs

Buffer:

PNK wash buffer: 20 mM Tris (pH 7.4), 10 mM $MgCl_2$, 0.2% Tween 20

1. Prepare PNK master mix:

Processed RNA	6 µg
10× PNK buffer	3 µl
PNK	1.5 µl
RiboLock (40 U/µl)	1 µl
Turbo DNase (2 U/µl)	1 µl
**Add DEPC-treated H_2O to**	**30 µl**

2. Incubate at 37 °C for 20 min.
3. Add 1 µl of 0.5 M EDTA (to chelate Mg^{2+} in the PNK buffer), heat it in a thermomixer at 75 °C for 10 min to inactivate the DNase and then chill on ice.
4. Add 0.5 µl of 1 M $MgCl_2$ to optimize the buffer for the subsequent ligation reaction.

Ligation I: Biotinylated 3' adapter ligation

1. Prepare Ligation I master mix:

DEPC-treated H_2O	2.5 µl
Input RNA	5 µl (1 µg)
DMSO (100%)	2.5 µl
10× T4 RNA ligase buffer	3 µl

Biotinylated 3' adapter (20 μM)	1 μl
50% PEG 8000 (wt/vol)	12 μl
RiboLock (40 U/μl)	1 μl
T4 RNA Ligase 1 (30 U/μl)	3 μl
**Total**	**30 μl**

2. Incubate the reaction in a thermomixer at 25 °C for 2 hours with shaking for 15 sec every 2 min.

Removal of free adapters

1. Add 31 μl of H_2O, 7 μl of 10× NEBuffer 2 (NEB, B7002S), and 2 μl of 5' deadenylase (50 U/μl, NEB, M0331S) to the Ligation I mix and incubate it in a thermomixer at 30 °C for 20 min with shaking for 15 sec every 2 min.
2. Add 4 μl of H_2O, 1 μl of 10× NEBuffer 2, and 5 μl of RecJ (30 U/μl, NEB, M0264S) and incubate it in a thermomixer at 37 °C for 30 min with shaking for 15 sec every 2 min.

RNA recovery by Streptavidin beads

Buffers:

Streptavidin pull-down buffer: 10 mM Tris (pH 7.4), 1 M NaCl, 1 mM EDTA, 0.2% Tween 20

Streptavidin wash buffer: 10 mM Tris (pH 7.4), 0.2% Tween 20

1. Dilute the ligation mixture in 190 μl of Streptavidin pull-down buffer.
2. Add 0.3 mg of pre-washed Streptavidin beads resuspended in 30 μl of Streptavidin pull-down buffer to the diluted ligation mixture.
3. Rotate the mixture at 25 °C for 20 min.
4. Wash the beads with 1 ml of Streptavidin pull-down buffer for 3× 5 min.
5. Wash the beads twice with 1 ml of Streptavidin wash buffer.

All the following steps are the same as SpyCLIP except that only one tube of an 80-μl PCR mixture is prepared and 10 cycles (6 three-step cycles and 4 two-step cycles) are performed to amplify the input cDNA library.

Materials used in the buffers

1 M Tris (pH 7.4): Sigma-Aldrich, T2194

5 M NaCl: Sigma-Aldrich, S5150

0.5 M EDTA (pH 8.0): Sigma-Aldrich, E7889

1 M DTT: Sigma-Aldrich, 43816

1 M $MgCl_2$: Sigma-Aldrich, M1028

Triton X-100: Sigma Aldrich, T8787

Tween 20: Sigma-Aldrich, P9416

30% BSA: Amresco, K719

Sodium dodecyl sulfate: Sigma-Aldrich, L3771

Sodium deoxycholate: Sigma-Aldrich, D6750

Urea: Sigma-Aldrich, 51456

Oligos used in this protocol

Biotinylated 3' adapter:

5'rApp-TGGAATTCTCGGGTGCCAAGG-3'biotin

UMI 3' adapter:

5'Phos-NNNNNNNNNNGATCGTCGGACTGTAGAACTCTGAAC-3'ddC

RT primer:

5'-CCTTGGCACCCGAGAATTCCA-3'

RP1 and RPI PCR primers are the same as those used in the Illumina TruSeq Small RNA kit and can be found at:

https://support.illumina.com/content/dam/illumina-support/documents/documentation/chemistry_documentation/experiment-design/illumina-adapter-sequences-1000000002694-06.pdf

Supplementary Sequence information

DNA and amino acid sequences of RBPs fused in frame with SpyTag

RBFOX2

ATGGACTACAAAGACCATGACGGTGATTATAAAGATCATGACATCGACTACAAGGATGACGATGACAA
GGGTTCAGGCCTGGAAGTTCTGTTCCAGGGGCCCGGAAGCGGTGCCCACATCGTGATGGTGGACGCCT
ACAAGCCGACGAAGGGCGGAGGAGGATCCATGGAGAAAAAGAAAATGGTAACTCAGGGTAACCAGGAG
CCGACAACAACTCCTGACGCAATGGTTCAGCCTTTTACTACCATCCCATTTCCACCACCTCCGCAGAA
TGGAATTCCCACAGAGTATGGGGTGCCACACACTCAAGACTATGCCGGCCAGACCGGTGAGCATAACC
TGACACTCTACGGAAGTACGCAAGCCCACGGGGAGCAGAGCAGCAACTCACCCAGCACACAAAATGGA
TCTCTTACGACAGAAGGTGGAGCACAGACAGACGGCCAGCAGTCACAGACACAAAGTAGTGAAAATTC
AGAGAGTAAATCTACCCCGAAACGGCTGCATGTCTCTAATATTCCTTTCCGCTTCCGGGACCCTGACC
TCCGGCAGATGTTTGGGCAGTTTGGCAAAATCCTAGATGTAGAAATAATCTTTAATGAACGTGGCTCT
AAGGGATTCGGGTTCGTAACTTTCGAGAATAGTGCTGATGCAGACAGGGCCAGGGAGAAATTACACGG
CACCGTGGTAGAGGGCCGTAAAATCGAGGTGAATAATGCTACAGCACGTGTAATGACCAATAAGAAGA
TGGTCACACCATATGCAAATGGTTGGAAATTAAGCCCAGTAGTTGGAGCTGTATATGGTCCGGAGTTA
TATGCAGCATCCAGCTTTCAAGCAGATGTGTCCCTAGGCAATGATGCAGCAGTGCCCCTATCAGGAAG
AGGGGGTATCAACACTTACATTCCTTTAATCAGTCTCCCTTTAGTTCCTGGCTTCCCTTACCCTACTG
CAGCCACCACGGCAGCCGCTTTCAGAGGAGCCCATTTGAGGGGCAGAGGGCGGACAGTATATGGTGCA
GTCCGAGCGGTACCTCCAACAGCCATCCCCGCCTATCCAGGTGTGGTTTACCAGGACGGATTTTACGG
TGCTGACCTCTATGGTGGATATGCAGCCTACAGATATGCACAGCCTGCTACTGCAACCGCAGCCACCG
CTGCTGCAGCCGCTGCAGCCGCTTACAGTGACGGTTATGGCAGGGTGTACACAGCCGACCCCTACCAT
GCCCTTGCCCCTGCCGCTAGCTATGGAGTTGGCGCTGTGGCGAGTTTATACCGAGGTGGCTACAGCCG
ATTTGCCCCCTACTGA

MDYKDHDGDYKDHDIDYKDDDDKGSGLEVLFQGPGSGAHIVMVDAYKPTKGGGGSMEKKKMVTQGNQE
PTTTPDAMVQPFTTIPFPPPPQNGIPTEYGVPHTQDYAGQTGEHNLTLYGSTQAHGEQSSNSPSTQNG
SLTTEGGAQTDGQQSQTQSSENSESKSTPKRLHVSNIPFRFRDPDLRQMFGQFGKILDVEIIFNERGS
KGFGFVTFENSADADRAREKLHGTVVEGRKIEVNNATARVMTNKKMVTPYANGWKLSPVVGAVYGPEL
YAASSFQADVSLGNDAAVPLSGRGGINTYIPLISLPLVPGFPYPTAATTAAAFRGAHLRGRGRTVYGA
VRAVPPTAIPAYPGVVYQDGFYGADLYGGYAAYRYAQPATATAATAAAAAAAAYSDGYGRVYTADPYH
ALAPAASYGVGAVASLYRGGYSRFAPY

SLBP

ATGGACTACAAAGACCATGACGGTGATTATAAAGATCATGACATCGACTACAAGGATGACGATGACAA
GGGTTCAGGCCTGGAAGTTCTGTTCCAGGGGCCCGGAAGCGGTGCCCACATCGTGATGGTGGACGCCT
ACAAGCCGACGAAGGGCGGAGGAGGATCCATGGCCTGCCGCCCGCGAAGCCCGCCGAGGCATCAGAGC
CGCTGCGACGGTGACGCCAGCCCGCCGTCCCCCGCGCGATGGAGCCTGGGACGGAAGCGCAGAGCCGA

CGGCAGGCGCTGGAGGCCCGAAGACGCCGAGGAGGCAGAGCACCGCGGCGCCGAGCGCAGACCCGAGA
GCTTTACCACTCCTGAAGGCCCTAAACCCCGTTCCAGATGCTCTGACTGGGCAAGTGCAGTTGAAGAA
GATGAAATGAGGACCAGAGTTAACAAAGAAATGGCAAGATATAAAAGGAAACTCCTCATCAATGACTT
TGGAAGAGAGAGAAAATCATCATCAGGAAGTTCTGATTCAAAGGAGTCTATGTCTACTGTGCCGGCTG
ACTTTGAGACAGATGAAAGTGTCCTAATGAGGAGACAGAAGCAGATCAACTATGGGAAGAACACAATT
GCCTACGATCGTTATATTAAAGAAGTCCCAAGACACCTTCGACAACCTGGCATTCATCCCAAGACCCC
TAATAAATTTAAGAAGTATAGTCGACGTTCATGGGACCAGCAAATCAAACTCTGGAAGGTGGCTCTGC
ATTTTTGGGACCCTCCAGCGGAAGAAGGATGTGATTTGCAAGAAATACACCCTGTAGACCTTGAATCT
GCAGAAAGCAGCTCCGAGCCCCAGACCAGCTCTCAGGATGACTTTGATGTGTACTCTGGCACACCCAC
CAAGGTGAGACACATGGACAGTCAAGTGGAGGATGAGTTTGATTTGGAAGCTTGTTTAACTGAACCCT
TGAGAGACTTCTCAGCCATGAGCTAA

MDYKDHDGDYKDHDIDYKDDDDKGSGLEVLFQGPGSGAHIVMVDAYKPTKGGGGSMACRPRSPPRHQS
RCDGDASPPSPARWSLGRKRRADGRRWRPEDAEEAEHRGAERRPESFTTPEGPKPRSRCSDWASAVEE
DEMRTRVNKEMARYKRKLLINDFGRERKSSSGSSDSKESMSTVPADFETDESVLMRRQKQINYGKNTI
AYDRYIKEVPRHLRQPGIHPKTPNKFKKYSRRSWDQQIKLWKVALHFWDPPAEEGCDLQEIHPVDLES
AESSSEPQTSSQDDFDVYSGTPTKVRHMDSQVEDEFDLEACLTEPLRDFSAMS

AGO2

ATGGACTACAAAGACCATGACGGTGATTATAAAGATCATGACATCGACTACAAGGATGACGATGACAA
GGGTTCAGGCCTGGAAGTTCTGTTCCAGGGGCCCGGAAGCGGTGCCCACATCGTGATGGTGGACGCCT
ACAAGCCGACGAAGGGCGGAGGAGGATCCATGTACTCGGGAGCCGGCCCCGCACTTGCACCTCCTGCG
CCGCCGCCCCCCATCCAAGGATATGCCTTCAAGCCTCCACCTAGACCCGACTTTGGGACCTCCGGGAG
AACAATCAAATTACAGGCCAATTTCTTCGAAATGGACATCCCCAAAATTGACATCTATCATTATGAAT
TGGATATCAAGCCAGAGAAGTGCCCGAGGAGAGTTAACAGGGAAATCGTGGAACACATGGTCCAGCAC
TTTAAAACACAGATCTTTGGGGATCGGAAGCCCGTGTTTGACGGCAGGAAGAATCTATACACAGCCAT
GCCCCTTCCGATTGGGAGGGACAAGGTGGAGCTGGAGGTCACGCTGCCAGGAGAAGGCAAGGATCGCA
TCTTCAAGGTGTCCATCAAGTGGGTGTCCTGCGTGAGCTTGCAGGCGTTACACGATGCACTTTCAGGG
CGGCTGCCCAGCGTCCCTTTTGAGACGATCCAGGCCCTGGACGTGGTCATGAGGCACTTGCCATCCAT
GAGGTACACCCCCGTGGGCCGCTCCTTCTTCACCGCGTCCGAAGGCTGCTCTAACCCTCTTGGCGGGG
GCCGAGAAGTGTGGTTTGGCTTCCATCAGTCCGTCCGGCCTTCTCTCTGGAAAATGATGCTGAATATT
GATGTGTCAGCAACAGCGTTTTACAAGGCACAGCCAGTAATCGAGTTTGTTTGTGAAGTTTTGGATTT
TAAAAGTATTGAAGAACAACAAAAACCTCTGACAGATTCCCAAAGGGTAAAGTTTACCAAAGAAATTA
AAGGTCTAAAGGTGGAGATAACGCACTGTGGGCAGATGAAGAGGAAGTACCGCGTCTGCAATGTGACC
CGGCGGCCCGCCAGTCACCAAACATTCCCGCTGCAGCAGGAGAGCGGGCAGACGGTGGAGTGCACGGT
GGCCCAGTATTTCAAGGACAGGCACAAGTTGGTTCTGCGCTACCCCCACCTCCCATGTTTACAAGTCG
GACAGGAGCAGAAACACACCTACCTTCCCCTGGAGGTCTGTAACATTGTGGCAGGACAAAGATGTATT
AAAAAATTAACGGACAATCAGACCTCAACCATGATCAGAGCGACTGCTAGGTCGGCGCCCGATCGGCA
AGAAGAGATTAGCAAATTGATGCGAAGTGCAAGTTTCAACACAGATCCATACGTCCGTGAATTTGGAA
TCATGGTCAAAGATGAGATGACAGACGTGACTGGGCGGGTGCTGCAGCCGCCCTCCATCCTCTACGGG
GGCAGGAATAAAGCTATTGCGACCCCTGTCCAGGGCGTCTGGGACATGCGGAACAAGCAGTTCCACAC

GGGCATCGAGATCAAGGTGTGGGCCATTGCGTGCTTCGCCCCCCAGCGCCAGTGCACGGAAGTCCATC
TGAAGTCCTTCACAGAGCAGCTCAGAAAGATCTCGAGAGACGCCGGCATGCCCATCCAGGGCCAGCCG
TGCTTCTGCAAATACGCGCAGGGGGCGGACAGCGTGGAGCCCATGTTCCGGCACCTGAAGAACACGTA
TGCGGGCCTGCAGCTGGTGGTGGTCATCCTGCCCGGCAAGACGCCCGTGTACGCCGAGGTCAAGCGCG
TGGGAGACACGGTGCTGGGGATGGCCACGCAGTGCGTGCAGATGAAGAACGTGCAGAGGACCACGCCA
CAGACCCTGTCCAACCTCTGCCTGAAGATCAACGTCAAGCTGGGAGGCGTGAACAACATCCTGCTGCC
CCAGGGCAGGCCGCCGGTGTTCCAGCAGCCCGTCATCTTTCTGGGAGCAGACGTCACTCACCCCCCCG
CCGGGGATGGGAAGAAGCCCTCCATTGCCGCCGTGGTGGGCAGCATGGACGCCCACCCCAATCGCTAC
TGCGCCACCGTGCGCGTGCAGCAGCACCGGCAGGAGATCATACAAGACCTGGCCGCCATGGTCCGCGA
GCTCCTCATCCAGTTCTACAAGTCCACGCGCTTCAAGCCCACCCGCATCATCTTCTACCGCGACGGTG
TCTCTGAAGGCCAGTTCCAGCAGGTTCTCCACCACGAGTTGCTGGCCATCCGTGAGGCCTGTATCAAG
CTAGAAAAAGACTACCAGCCCGGGATCACCTTCATCGTGGTGCAGAAGAGGCACCACACCCGGCTCTT
CTGCACTGACAAGAACGAGCGGGTTGGGAAAAGTGGAAACATTCCAGCAGGCACGACTGTGGACACGA
AAATCACCCACCCCACCGAGTTCGACTTCTACCTGTGTAGTCACGCTGGCATCCAGGGGACAAGCAGG
CCTTCGCACTATCACGTCCTCTGGGACGACAATCGTTTCTCCTCTGATGAGCTGCAGATCCTAACCTA
CCAGCTGTGTCACACCTACGTGCGCTGCACACGCTCCGTGTCCATCCCAGCGCCAGCATACTACGCTC
ACCTGGTGGCCTTCCGGGCCAGGTACCACCTGGTGGATAAGGAACATGACAGTGCTGAAGGAAGCCAT
ACCTCTGGGCAGAGTAACGGGCGAGACCACCAAGCACTGGCCAAGGCGGTCCAGGTTCACCAAGACAC
TCTGCGCACCATGTACTTTGCTTGA

MDYKDHDGDYKDHDIDYKDDDDKGSGLEVLFQGPGSGAHIVMVDAYKPTKGGGGSMYSGAGPALAPPA
PPPPIQGYAFKPPPRPDFGTSGRTIKLQANFFEMDIPKIDIYHYELDIKPEKCPRRVNREIVEHMVQH
FKTQIFGDRKPVFDGRKNLYTAMPLPIGRDKVELEVTLPGEGKDRIFKVSIKWVSCVSLQALHDALSG
RLPSVPFETIQALDVVMRHLPSMRYTPVGRSFFTASEGCSNPLGGGREVWFGFHQSVRPSLWKMMLNI
DVSATAFYKAQPVIEFVCEVLDFKSIEEQQKPLTDSQRVKFTKEIKGLKVEITHCGQMKRKYRVCNVT
RRPASHQTFPLQQESGQTVECTVAQYFKDRHKLVLRYPHLPCLQVGQEQKHTYLPLEVCNIVAGQRCI
KKLTDNQTSTMIRATARSAPDRQEEISKLMRSASFNTDPYVREFGIMVKDEMTDVTGRVLQPPSILYG
GRNKAIATPVQGVWDMRNKQFHTGIEIKVWAIACFAPQRQCTEVHLKSFTEQLRKISRDAGMPIQGQP
CFCKYAQGADSVEPMFRHLKNTYAGLQLVVVILPGKTPVYAEVKRVGDTVLGMATQCVQMKNVQRTTP
QTLSNLCLKINVKLGGVNNILLPQGRPPVFQQPVIFLGADVTHPPAGDGKKPSIAAVVGSMDAHPNRY
CATVRVQQHRQEIIQDLAAMVRELLIQFYKSTRFKPTRIIFYRDGVSEGQFQQVLHHELLAIREACIK
LEKDYQPGITFIVVQKRHHTRLFCTDKNERVGKSGNIPAGTTVDTKITHPTEFDFYLCSHAGIQGTSR
PSHYHVLWDDNRFSSDELQILTYQLCHTYVRCTRSVSIPAPAYYAHLVAFRARYHLVDKEHDSAEGSH
TSGQSNGRDHQALAKAVQVHQDTLRTMYFA

PTBP1

ATGGACTACAAAGACCATGACGGTGATTATAAAGATCATGACATCGACTACAAGGATGACGATGACAA
GGGTTCAGGCCTGGAAGTTCTGTTCCAGGGGCCCGGAAGCGGTGCCCACATCGTGATGGTGGACGCCT
ACAAGCCGACGAAGGGCGGAGGAGGATCCATGGACGGCATTGTCCCAGATATAGCCGTTGGTACAAAG
CGGGGATCTGACGAGCTTTTCTCTACTTGTGTCACTAACGGACCGTTTATCATGAGCAGCAACTCGGC
TTCTGCAGCAAACGGAAATGACAGCAAGAAGTTCAAAGGTGACAGCCGAAGTGCAGGCGTCCCCTCTA

GAGTGATCCACATCCGGAAGCTCCCCATCGACGTCACGGAGGGGGAAGTCATCTCCCTGGGGCTGCCC
TTTGGGAAGGTCACCAACCTCCTGATGCTGAAGGGGAAAAACCAGGCCTTCATCGAGATGAACACGGA
GGAGGCTGCCAACACCATGGTGAACTACTACACCTCGGTGACCCCTGTGCTGCGCGGCCAGCCCATCT
ACATCCAGTTCTCCAACCACAAGGAGCTGAAGACCGACAGCTCTCCCAACCAGGCGCGGGCCCAGGCG
GCCCTGCAGGCGGTGAACTCGGTCCAGTCGGGGAACCTGGCCTTGGCTGCCTCGGCGGCGGCCGTGGA
CGCAGGGATGGCGATGGCCGGGCAGAGCCCCGTGCTCAGGATCATCGTGGAGAACCTCTTCTACCCTG
TGACCCTGGATGTGCTGCACCAGATTTTCTCCAAGTTCGGCACAGTGTTGAAGATCATCACCTTCACC
AAGAACAACCAGTTCCAGGCCCTGCTGCAGTATGCGGACCCCGTGAGCGCCCAGCACGCCAAGCTGTC
GCTGGACGGGCAGAACATCTACAACGCCTGCTGCACGCTGCGCATCGACTTTTCCAAGCTCACCAGCC
TCAACGTCAAGTACAACAATGACAAGAGCCGTGACTACACACGCCCAGACCTGCCTTCCGGGGACAGC
CAGCCCTCGCTGGACCAGACCATGGCCGCGGCCTTCGGTGCACCTGGTATAATCTCAGCCTCTCCGTA
TGCAGGAGCTGGTTTCCCTCCCACCTTTGCCATTCCTCAAGCTGCAGGCCTTTCCGTTCCGAACGTCC
ACGGCGCCCTGGCCCCCCTGGCCATCCCCTCGGCGGCGGCGGCAGCTGCGGCGGCAGGTCGGATCGCC
ATCCCGGGCCTGGCGGGGGCAGGAAATTCTGTATTGCTGGTCAGCAACCTCAACCCAGAGAGAGTCAC
ACCCCAAAGCCTCTTTATTCTTTTCGGCGTCTACGGTGACGTGCAGCGCGTGAAGATCCTGTTCAATA
AGAAGGAGAACGCCCTAGTGCAGATGGCGGACGGCAACCAGGCCCAGCTGGCCATGAGCCACCTGAAC
GGGCACAAGCTGCACGGGAAGCCCATCCGCATCACGCTCTCGAAGCACCAGAACGTGCAGCTGCCCCG
CGAGGGCCAGGAGGACCAGGGCCTGACCAAGGACTACGGCAACTCACCCCTGCACCGCTTCAAGAAGC
CGGGCTCCAAGAACTTCCAGAACATATTCCCGCCCTCGGCCACGCTGCACCTCTCCAACATCCCGCCC
TCAGTCTCCGAGGAGGATCTCAAGGTCCTGTTTTCCAGCAATGGGGGCGTCGTCAAAGGATTCAAGTT
CTTCCAGAAGGACCGCAAGATGGCACTGATCCAGATGGGCTCCGTGGAGGAGGCGGTCCAGGCCCTCA
TTGACCTGCACAACCACGACCTCGGGGAGAACCACCACCTGCGGGTCTCCTTCTCCAAGTCCACCATC
TAG

MDYKDHDGDYKDHDIDYKDDDDKGSGLEVLFQGPGSGAHIVMVDAYKPTKGGGGSMDGIVPDIAVGTK
RGSDELFSTCVTNGPFIMSSNSASAANGNDSKKFKGDSRSAGVPSRVIHIRKLPIDVTEGEVISLGLP
FGKVTNLLMLKGKNQAFIEMNTEEAANTMVNYYTSVTPVLRGQPIYIQFSNHKELKTDSSPNQARAQA
ALQAVNSVQSGNLALAASAAAVDAGMAMAGQSPVLRIIVENLFYPVTLDVLHQIFSKFGTVLKIITFT
KNNQFQALLQYADPVSAQHAKLSLDGQNIYNACCTLRIDFSKLTSLNVKYNNDKSRDYTRPDLPSGDS
QPSLDQTMAAAFGAPGIISASPYAGAGFPPTFAIPQAAGLSVPNVHGALAPLAIPSAAAAAAAAGRIA
IPGLAGAGNSVLLVSNLNPERVTPQSLFILFGVYGDVQRVKILFNKKENALVQMADGNQAQLAMSHLN
GHKLHGKPIRITLSKHQNVQLPREGQEDQGLTKDYGNSPLHRFKKPGSKNFQNIFPPSATLHLSNIPP
SVSEEDLKVLFSSNGGVVKGFKFFQKDRKMALIQMGSVEEAVQALIDLHNHDLGENHHLRVSFSKSTI
